# Rural-to-urban migrant worker mobility shaped measles epidemics in China

**DOI:** 10.1371/journal.pcbi.1014182

**Published:** 2026-04-10

**Authors:** Peihua Wang, Xianwen Wang, Wenyi Zhang, Yong Wang, Sen Pei, Xiao-Ke Xu, Wan Yang

**Affiliations:** 1 Department of Epidemiology, Mailman School of Public Health, Columbia University, New York, New York, United States of America; 2 School of Public Administration and Policy, Dalian University of Technology, Dalian, China; 3 Chinese PLA Center for Disease Control and Prevention, Beijing, China; 4 Department of Environmental Health Sciences, Mailman School of Public Health, Columbia University, New York, New York, United States of America; 5 School of Journalism and Communication, Beijing Normal University, Beijing, China; Fundação Getúlio Vargas: Fundacao Getulio Vargas, BRAZIL

## Abstract

Despite sustained high routine childhood vaccination coverage, measles outbreaks have persisted across Provincial-Level Administrative Divisions (PLADs) in China. Epidemiological evidence suggests that migrant workers substantially contribute to these outbreaks. In this study, we investigated the role of inter-PLAD rural-to-urban migrant workers, who originate from less developed rural regions with potentially lower vaccination coverage and are employed in urban centers, in contributing to measles epidemics in China from 2005 to 2014. We developed a networked metapopulation Susceptible–Exposed–Infectious–Recovered model that incorporated migrant worker mobility around Chinese New Year (CNY) migration periods and year-round general-purpose traveler mobility. By simulating measles transmission dynamics within migrant worker subpopulations, we identified key epidemiological connections between origin and host PLADs. In northern China, migrant workers from Hebei and Shandong were the key contributors to outbreaks in two northern host PLADs, Beijing and Tianjin. In southern China, migrant workers from Anhui and Sichuan were the key contributors across multiple southern host PLADs. Counterfactual modeling suggests that measles epidemics in host PLADs were sustained by susceptibility replenishment through inflows of under-vaccinated migrant workers during the CNY migration periods. Moreover, epidemics in origin PLADs might have been synchronized and facilitated by case importation of exposed and infectious migrant workers returning from endemic host PLADs, and the strength of this seeding effect depended on the volume of migrant worker flows. Traveler mobility showed minimal impact on measles epidemics. Counterfactual modeling of pre-migration vaccination with coverage ranging from 25% to 100% showed national incidence reduction from 33.0% to 50.9%, with significant reduction in host PLADs, and in turn in origin PLADs due to weakened seeding effect. Our findings provide mechanistic insights into the epidemiological role of rural-to-urban migrant workers in measles epidemics, which could support targeted vaccination strategies for improved measles control in China and regions with similar migration dynamics.

## Author summary

Measles transmission persisted across Provincial-Level Administrative Divisions (PLADs) in China from 2005 to 2014 despite high routine childhood vaccination coverage. We tested our hypothesis that the mobility of inter-PLAD rural-to-urban migrant workers contributed to measles outbreaks. These migrant workers (5.7% of the national population during the study period), often from less developed rural regions with potentially lower vaccination coverage, sought employment in urban centers, with the largest mobility flows occurring around the Chinese New Year. We found that migrant worker mobility shaped epidemics through two mechanisms. Epidemics in migrant worker host PLADs (urban destination regions) were sustained by inflows of under-vaccinated migrant workers that increased the proportion of people susceptible to measles. Epidemics in origin PLADs (rural home regions) might have been synchronized and facilitated by infected or exposed migrant workers returning from endemic host PLADs. In counterfactual simulations, vaccinating migrant workers before migration with coverage ranging from 25% to 100% reduced national incidence by 33.0% to 50.9%. These results suggest that targeted vaccination for migrant workers could strengthen measles control in China.

## Introduction

Measles is a highly infectious disease with an estimated basic reproductive number ($R_{\text{0}}$; i.e., average number of secondary infections caused by one infected individual in a fully susceptible population) of 12–18 [1,2]. Vaccination in children has been the most effective public health intervention, and models predict that at least 95% vaccination coverage is needed for measles elimination given its $R_{\text{0}}$ [3,4]. In China, routine measles vaccination was included in the national Expanded Program on Immunization (EPI) in 1978, and a two-dose schedule was introduced in 1986 [5]. However, despite reported routine childhood vaccination coverage exceeding 95% since 2005 in Provincial-Level Administrative Divisions (PLADs) such as Beijing [6], Tianjin [7], and other economically developed PLADs [5], annual measles outbreaks persisted through 2010. The nationwide Supplementary Immunization Activity (SIA) in 2010, targeting children aged 1–14 years, temporally curbed transmission, but outbreaks rebounded between 2013 and 2016. These immunization programs substantially reduced measles transmission among children, which was likely the primary driver of outbreaks when vaccination coverage was not sufficiently high [8]. Nevertheless, epidemiological studies in China have documented a shift towards increased prevalence among adults, particularly migrant workers originating from less developed regions [9–11]. These migrant workers generally have higher population susceptibility due to limited healthcare accessibility and consequently lower vaccination coverage in their hometowns [12,13]. Modeling studies in China [14,15] further support these epidemiological findings, suggesting that in addition to climate factors modulating measles transmission, migrant worker mobility contributes to measles outbreaks by replenishing susceptible population in host PLADs.

Migrant workers constitute a substantial portion of the Chinese population (19.6% of total population in 2010 [16]) and primarily comprise two types: rural-to-urban migrant workers and urban-origin white-collar migrant workers. Rural-to-urban migrant workers originate from rural areas (i.e., holding rural household registrations within the Chinese household registration system, which officially identifies individuals as permanent residents of specific locations and determines their access to local social welfare, healthcare, and educational benefits [17]). They migrate to urban areas and are predominantly employed in manufacturing, construction, wholesale, and retail sectors [18]. In contrast, urban-origin white-collar migrant workers typically have higher education levels and are employed in professional and managerial positions within urban centers [19]. Due to lower economic development and limited healthcare accessibility in their rural hometowns, combined with limited healthcare benefits associated with rural household registrations, rural-to-urban migrant workers likely have lower measles vaccination coverage compared to white-collar migrant workers, and thus may play a crucial role in measles epidemics.

From 2005 to 2014, inter-PLAD rural-to-urban migrant workers (hereafter referred to as migrant workers) averaged 75 million annually, accounting for 5.7% of the national population in China [20]. This population typically migrated to economically developed PLADs following the Chinese New Year (CNY) holiday to seek employment. Such large-scale seasonal migration is analogous to migration driven by agricultural cycles, which has been shown to shape measles epidemics in Africa [21,22], and could rapidly alter the contact patterns and population immunological profiles in host PLADs. Notably, this migrant worker influx temporally coincided with epidemic peaks around April [15]. Given the magnitude and timing of worker migration, detailed mechanistic modeling of rural-to-urban migrant worker mobility is needed to quantitatively assess its impact on measles epidemics.

In this study, we investigated the role of inter-PLAD rural-to-urban migrant workers in contributing to measles epidemics in host PLADs from 2005 to 2014. We developed a networked metapopulation Susceptible–Exposed–Infectious–Recovered (SEIR) model that incorporated migrant worker mobility around CNY migration periods and year-round general-purpose traveler mobility. Using this model, we simulated transmission dynamics within migrant worker subpopulations and identified key epidemiological connections between origin and host PLADs. We further quantified the impacts of migrant worker mobility, traveler mobility, and population immunological profiles on measles epidemics. Our results provide mechanistic insights into the epidemiological role of rural-to-urban migrant workers in measles epidemics, which could inform targeted public health interventions to control measles transmission in China.

## Results

### Mobility patterns and their associations with measles incidence

**Fig 1:**
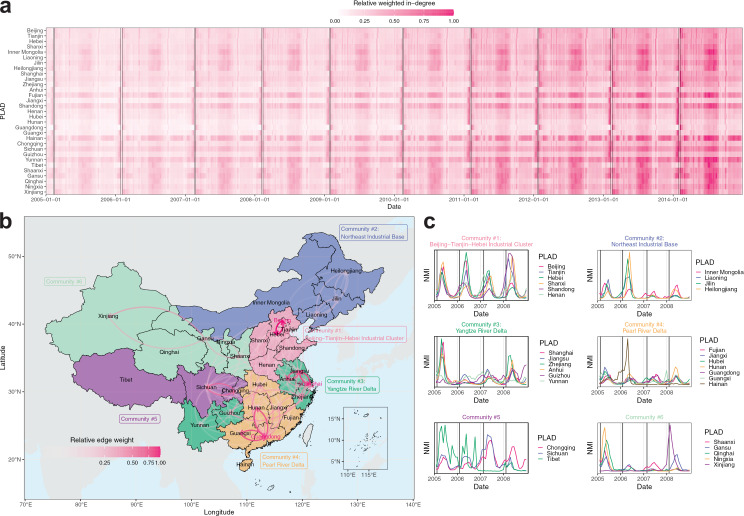
Mobility patterns and their associations with measles incidence. **(a)** Relative weighted in-degree of the inter-PLAD traveler network from 2005 to 2014. Boxes indicate CNY holiday periods. **(b)** Infomap community detection based on aggregated mobility patterns six weeks before and after the CNY holidays. Directed edges indicate mobility flows, and PLADs highlighted in red indicate transportation hubs. **(c)** Normalized monthly incidence (NMI) of measles in PLADs within each community from 2005 to 2008, a period characterized by regular annual outbreaks before the nationwide SIA in 2010. Vertical lines indicate the first day of each CNY holiday. PLAD and country boundary basemaps were obtained from the publicly available Natural Earth shapefiles (https://www.naturalearthdata.com/downloads/; terms of use: https://www.naturalearthdata.com/about/terms-of-use/), via the R packages rnaturalearth (v1.0.1) [24] and sf (v1.0-15) [25].

Time series of inter-PLAD traveler network (general-purpose travel, including tourism, business trips, and white-collar worker migration) from 2005 to 2014 was used to compute network characteristics of PLADs. PLADs such as Beijing, Tianjin, Shanghai, Jiangsu, Zhejiang, Fujian, and Guangdong showed increased population outflows before the CNY holidays (S1 Fig) and inflows afterward (Fig 1a). These PLADs had the highest per-capita Gross Regional Product (GRP) [20] and had the largest proportions of inter-PLAD rural-to-urban migrant workers (≥8.6%; S2 Fig), thus serving as migrant worker host PLADs. Conversely, less developed PLADs, home to most migrant workers, showed reversed mobility flows. These seasonal mobility patterns primarily resulted from migrant workers returning to their origin PLADs before the CNY holidays for family reunions and to host PLADs for employment afterward.

We constructed an aggregated traveler network by summing traveler volumes (edge weights) between each PLAD pair (node pair) over the migration periods (six weeks before and after the CNY holidays) from the original time-series traveler network. Infomap community detection [23] was then applied to partition the network by minimizing the description length of random walks, resulting in six communities characterized by dense internal traveler flows (Fig 1b). Four of these overlapped with key economic zones in China: the Beijing–Tianjin–Hebei Industrial Cluster (community #1) and the Northeast Industrial Base (historically a heavy industry center; community #2) in the north, the Yangtze River Delta (community #3; Zhejiang, Jiangsu, and Shanghai were leading destinations for migrant workers from Guizhou and Yunnan despite geographic discontinuity; see details in S1 Text “Estimation of inter-PLAD rural-to-urban migrant worker population sizes”) in the east, and the Pearl River Delta (community #4) in the south. Beijing (in community #1), Shanghai (in community #3), and Guangdong (in community #4) were major transportation hubs with the highest PageRank centralities, collectively accounting for 47.0% of all flows during the CNY migration periods.

During the study period, measles outbreaks occurred with varying patterns across PLADs. PLADs in the Beijing–Tianjin–Hebei Industrial Cluster and the Pearl River Delta showed annual outbreaks of similar magnitude (Fig 1c). In contrast, PLADs in the Northeast Industrial Base and the Yangtze River Delta also showed annual outbreaks but of differing magnitudes (Fig 1c), whereas PLADs in western China showed less synchronized outbreaks (Fig 1c). Nonetheless, measles incidence in PLADs within the same communities showed higher correlations than that between communities (Mantel test, *r* = 0.10, *P* = 0.014). Some PLADs, such as Guizhou and Yunnan (Yangtze River Delta) showed highly irregular outbreaks potentially due to mountainous terrain and limited economic development that impeded healthcare accessibility and thereby influenced case reporting [15].

### Modeled population dynamics and measles epidemic dynamics

**Fig 2:**
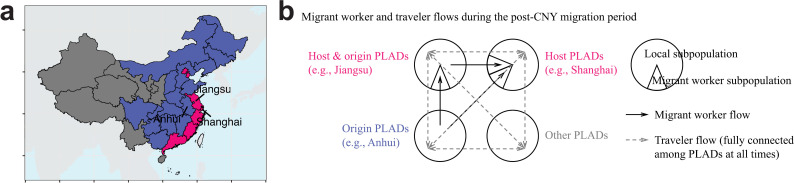
Schematic of the networked metapopulation SEIR model. **(a)** Migrant worker host PLADs (red), origin PLADs (blue), and PLADs that are neither host nor origin (gray), as defined based on national population sample surveys and per-capita GRP. **(b)** Schematic of migrant worker and traveler flows in the networked metapopulation SEIR model during the post-CNY migration period, illustrated using Jiangsu, Shanghai, and Anhui as an example. Migrant worker flow direction reverses during the pre-CNY migration period. No migrant worker flow is assumed during regular and CNY holiday periods. PLAD and country boundary basemaps were obtained from the publicly available Natural Earth shapefiles (https://www.naturalearthdata.com/downloads/; terms of use: https://www.naturalearthdata.com/about/terms-of-use/), via the R packages rnaturalearth (v1.0.1) [24] and sf (v1.0-15) [25].

The networked metapopulation SEIR model defined seven migrant worker host PLADs (Beijing, Tianjin, Shanghai, Jiangsu, Zhejiang, Fujian, and Guangdong; Fig 2a) based on their having the largest proportions of migrant workers and the highest per-capita GRP, and 18 migrant worker origin PLADs based on their population sizes within host PLADs (Fig 2a; see S2 Fig for detailed host–origin PLAD pairs). Two host PLADs, Jiangsu and Zhejiang, also served as origins for migrant workers. The remaining eight PLADs (Fig 2a), including western PLADs (Yunnan, Tibet, and the five PLADs in community #6, Fig 1b) and the island PLAD (Hainan), were neither host nor origin PLADs. The model integrated two mobility networks (Fig 2b): the traveler network (representing general-purpose travel), which was fully connected among PLADs and operated throughout the year, and the migrant worker network (representing rural-to-urban worker migration specifically), which connected host and origin PLADs and operated only during the migration periods (six weeks before and after the CNY holidays).

**Fig 3:**
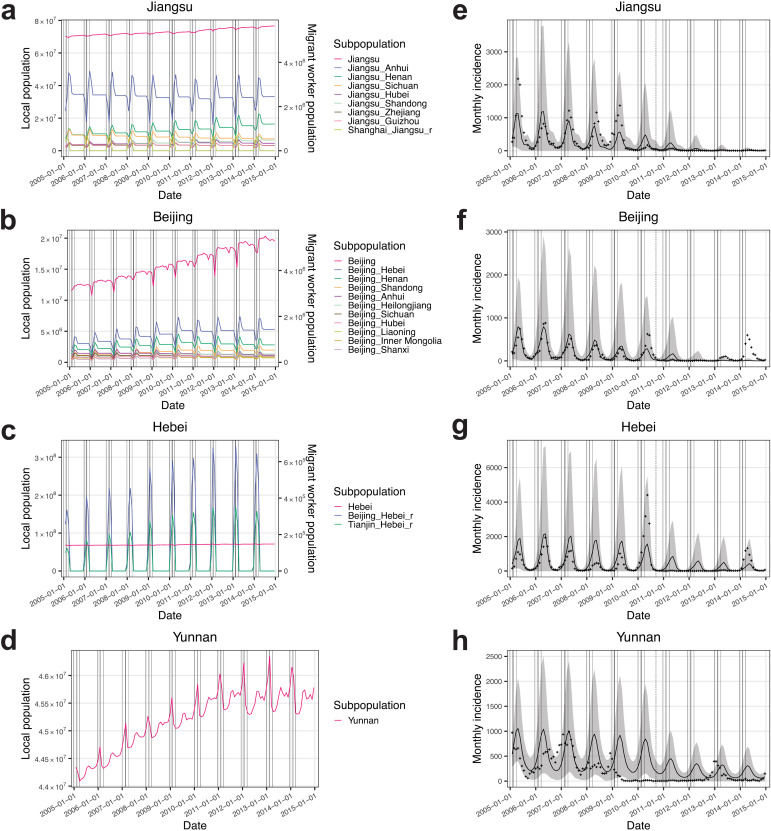
Modeled population dynamics and measles epidemic dynamics. Population dynamics of local and migrant worker subpopulations illustrated using examples from **(a)** Jiangsu (both a host and an origin PLAD), **(b)** Beijing (a host PLAD), **(c)** Hebei (an origin PLAD), and **(d)** Yunnan (neither a host nor an origin PLAD). Subpopulation labeled “PLAD A_PLAD B” indicates a migrant worker subpopulation with host PLAD A and origin PLAD B. Subpopulation labeled “PLAD A_PLAD B_r” indicates a returned migrant worker subpopulation with host PLAD A and origin PLAD B. Migrant worker subpopulations in the legend are ordered by their population sizes from the largest at the top to the smallest at the bottom. Vertical solid lines from left to right indicate the starts of pre-CNY, CNY, and post-CNY periods, and the end of post-CNY period for each year. Simulated measles incidence from the calibrated model ensemble for **(e)** Jiangsu, **(f)** Beijing, **(g)** Hebei, and **(h)** Yunnan, compared with observed incidence (crosses). Lines indicate median estimates, and shaded area indicates 95% confidence interval. The vertical dashed line indicates the nationwide SIA in 2010.

In host PLADs, such as Jiangsu (Fig 3a) and Beijing (Fig 3b), migrant worker populations (indicated as “host_origin”) tended to decrease during the 6-week pre-CNY period as migrant workers returned to the origin PLADs for family reunions, and increase during the 6-week post-CNY period as they moved into host PLADs for employment. Correspondingly, in origin PLADs, such as Jiangsu (Fig 3a) and Hebei (Fig 3c), migrant worker populations returning from host PLADs (indicated as “host_origin_r”, e.g., “Beijing_Hebei_r” in Hebei) showed dynamics opposite to their counterparts in host PLADs (e.g., “Beijing_Hebei” in Beijing). In the model, the migrant worker flow during the post-CNY period included both recurring and new migrant workers. Their populations in host PLADs peaked at the end of the post-CNY migration period and tended to decrease afterward (Fig 3a and 3b), as migrant workers who were unable to secure employment returned to their origin PLADs. PLADs that were neither host nor origin PLADs, such as Yunnan (Fig 3d), included only local populations. Local population dynamics in all PLADs included travel mobility, and both local and migrant worker population dynamics included births and deaths.

Incorporating the above population dynamics, we calibrated the model using monthly measles incidence data from 2005 to 2008 (a period characterized by regular annual outbreaks in China and before the nationwide SIA in 2010) for all PLADs (Figs 3e–3h and S3–S6). The calibrated model captured regular annual outbreaks and showed reasonable overall model fit (see PLAD-specific model fit metrics in S1 Table). Host PLADs and origin PLADs connected through the migrant worker network showed better model fits than the other disconnected western and island PLADs (comparison based on relative root mean square error (RRMSE), Mann–Whitney *U* test, *r* = 0.41 (95% CI: (0.03, 0.75)), *P* = 0.023; see RRMSE for each PLAD in S1 Table). These disconnected PLADs experienced irregular epidemic dynamics characterized by large outbreaks followed by epidemic fadeouts (Fig 1c), suggesting influences other than mobility, such as higher birth rates (Mann–Whitney *U* test, *r* = 0.57 (95% CI: (0.28, 0.76)), *P* = 0.001) and variability in vaccination coverage.

We further validated the model using out-of-sample incidence data from January 2009–September 2010 (i.e., prior to the nationwide SIA). Compared with the calibration period of 2005–2008, model performance declined (median RRMSE for the calibration and validation periods are 0.901 (IQR: (0.757, 1.558)) and 2.073 (IQR: (1.336, 5.521)), respectively; median correlation coefficients are 0.600 (IQR: (0.455, 0.743)) and 0.630 (IQR: (0.413, 0.712)), respectively; median peak time differences are 1.00 (IQR: (0.50, 1.50)) and 1.00 (IQR: (0.50, 1.50)) months, respectively; see PLAD-specific model validation metrics in S1 Table), potentially due to unsynchronized PLAD-level SIAs [26], which could not be incorporated into the model because of a lack of relevant data, potential changes in case reporting, and greater influence of stochastic fadeouts and case importations when incidence was very low. Additionally, mobility sensitivity analyses introducing flow variability produced similar simulated incidence time series between the base and alternative mobility scenarios, indicating robust model performance at the PLAD level (see PLAD-specific comparison metrics in S2 Table).

### Key migrant worker subpopulations contributing to measles outbreaks in host PLADs

**Fig 4:**
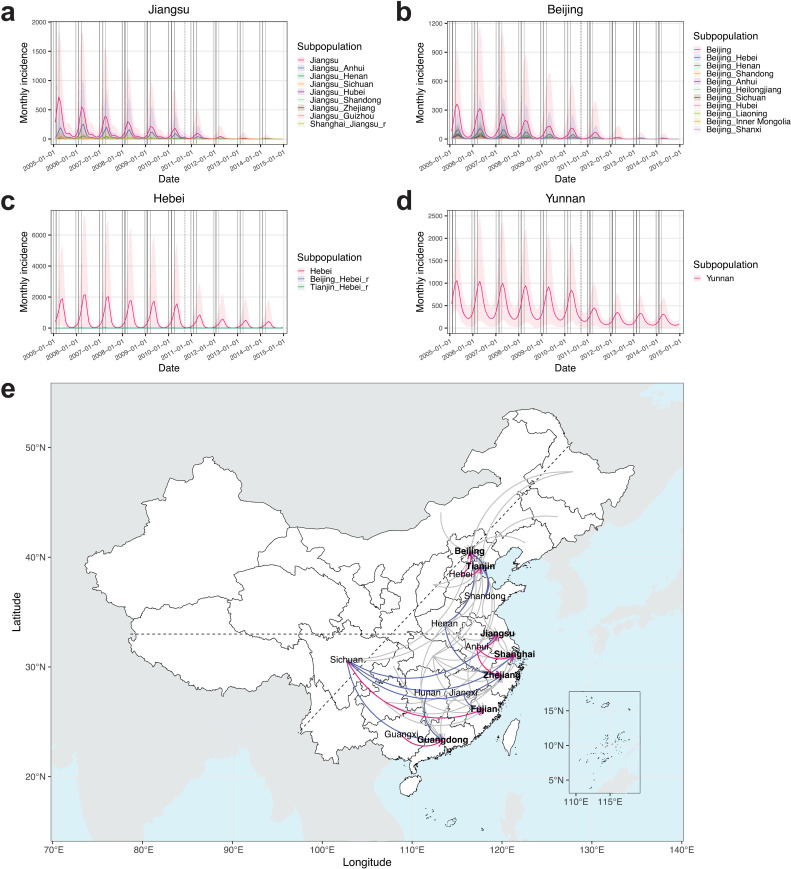
Key migrant worker subpopulations contributing to measles outbreaks in host PLADs. Simulated measles incidence from the calibrated model ensemble for each subpopulation, illustrated using examples from **(a)** Jiangsu, **(b)** Beijing, **(c)** Hebei, and **(d)** Yunnan. **(e)** Key epidemiological connections among PLADs. Arrows indicate migrant worker subpopulations moving from origin (tail) to host (head) PLADs. Arrows highlighted in red indicate migrant worker subpopulations that contributed the highest measles incidence among all migrant worker subpopulations in their corresponding host PLADs. Arrows highlighted in blue indicate additional migrant worker subpopulations that were among the top-ranked contributors to incidence in their corresponding host PLADs. Host PLADs are indicated in bold font. The horizontal dashed line approximates the Qin Mountains–Huai River reference line at ~33°N, which divides China into northern and southern regions, and the tiled dashed line indicates the Heihe–Tengchong reference line, which divides China into western and eastern regions. PLAD and country boundary basemaps were obtained from the publicly available Natural Earth shapefiles (https://www.naturalearthdata.com/downloads/; terms of use: https://www.naturalearthdata.com/about/terms-of-use/), via the R packages rnaturalearth (v1.0.1) [24] and sf (v1.0-15) [25].

The calibrated model ensemble simulated measles incidence for each subpopulation within PLADs (Fig 4a–4d). By ranking incidence within each host PLAD, we identified top-ranked migrant worker subpopulations that collectively accounted for >50% of total incidence from migrant workers (17 out of 52 in total, red arrows indicate the subpopulations contributing the highest incidence, and blue arrows indicate additional top-ranked subpopulations, Fig 4e). In northern China, Hebei and Shandong were key contributors to outbreaks in both northern host PLADs, namely Beijing and Tianjin (connections aligned with Infomap community #1, corresponding to the Beijing–Tianjin–Hebei Industrial Cluster, Fig 1b). In southern China, Anhui and Sichuan were the key contributors to outbreaks across multiple southern host PLADs. Specifically, Anhui was the primary contributor in Jiangsu, Shanghai, and Zhejiang (Infomap community #3, corresponding to the Yangtze River Delta, Fig 1b). Sichuan was the primary contributor in Fujian, and a key contributor across all the other southern host PLADs. It was the only western key PLAD, and it formed long-distance epidemiological connections to host PLADs. Additionally, Guangxi was the primary contributor in Guangdong (Infomap community #4, corresponding to the Pearl River Delta, Fig 1b). Mobility sensitivity analyses introducing flow variability identified the same primary epidemiological connections between the base and alternative mobility scenarios, with only a few additional connections shifting, involving origin PLADs Henan and Jiangxi (S7a and S7b Fig).

### Impacts of mobility and population immunological profiles assessed through counterfactual modeling

**Fig 5:**
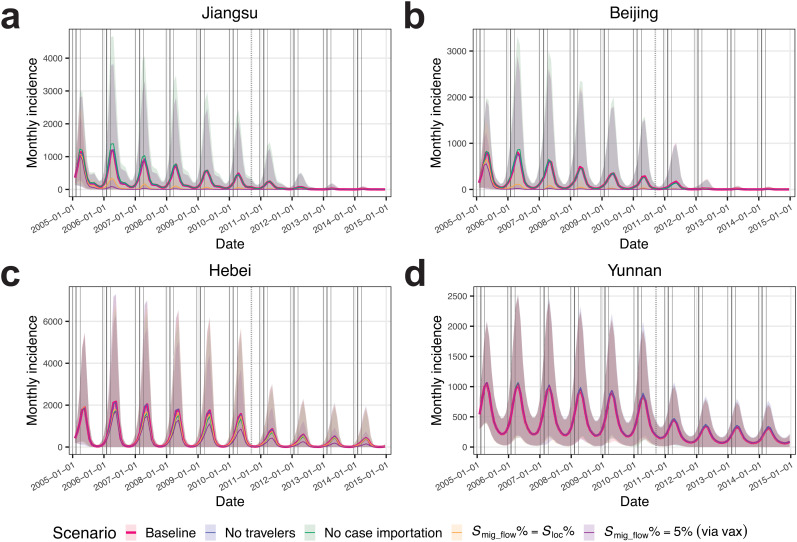
Impacts of mobility and population immunological profiles assessed through counterfactual modeling. Simulated measles incidence under the baseline scenario compared with that under counterfactual scenarios in **(a)** Jiangsu, **(b)** Beijing, **(c)** Hebei, and **(d)** Yunnan. Counterfactual scenarios include no travelers, no case importation, matching population susceptibility between incoming migrant workers and local populations in host PLADs, and pre-migration vaccination for migrant workers under 100% coverage.

We tested the impacts of traveler mobility (spatial spread) and migrant worker mobility (case importation of exposed and infected individuals and susceptibility replenishment through inflows of more susceptible populations) on measles epidemics by using counterfactual modeling and comparing the simulated cumulative incidence with that of the baseline scenario from the calibrated model ensemble. In the first analysis, we removed traveler mobility (“no travelers” scenario). The results showed minimal impact of non-migration-related general travel on measles epidemics (Tables 1 and S3 and Fig 5a–5d).

**Table 1:** Relative differences in cumulative measles incidence between the four different counterfactual scenarios and the baseline in host PLADs. Values indicate mean estimates with 95% confidence intervals (calculated using the bootstrap method).

China or host PLAD	Counterfactual scenario			
	No travelers	No case importation	Matching population susceptibility	Pre-migration vaccination (100% coverage)
China	-0.3% (-6.6%, 6.4%)	-15.4% (-21.9%, -8.5%)	-35.8% (-40.3%, -31.1%)	-50.9% (-54.2%, -47.2%)
Beijing	-0.7% (-19.1%, 19.7%)	6.4% (-15.2%, 31.9%)	-69.6% (-74.3%, -65.0%)	-79.1% (-82.2%, -76.0%)
Tianjin	0.6% (-22.9%, 26.8%)	0.0% (-22.5%, 25.6%)	-73.9% (-78.7%, -68.6%)	-83.9% (-86.8%, -80.8%)
Shanghai	-1.7% (-21.5%, 22.6%)	-22.7% (-41.1%, -0.2%)	-51.5% (-59.9%, -42.2%)	-73.7% (-78.1%, -69.0%)
Jiangsu	-0.6% (-20.8%, 23.1%)	3.8% (-18.4%, 30.6%)	-74.5% (-78.8%, -70.3%)	-84.0% (-86.6%, -81.3%)
Zhejiang	0.7% (-18.1%, 23.1%)	10.6% (-12.4%, 37.9%)	-72.6% (-76.6%, -67.6%)	-82.3% (-84.9%, -79.4%)
Fujian	1.8% (-27.2%, 37.0%)	1.8% (-28.3%, 39.3%)	-77.4% (-82.8%, -72.2%)	-84.8% (-88.1%, -81.1%)
Guangdong	-0.2% (-16.5%, 18.5%)	13.8% (-8.6%, 38.9%)	-69.9% (-75.3%, -64.1%)	-89.2% (-91.0%, -87.0%)

The second analysis, removing the movement of exposed and infectious migrant workers (“no case importation” scenario), estimated a statistically significant 15.4% reduction in national measles incidence, largely due to reductions in origin PLADs (Tables 1 and S3). Specifically, this scenario showed no significant change in incidence in host PLADs (see Jiangsu in Fig 5a and Beijing in Fig 5b as examples), except for Shanghai, where incidence decreased (Table 1). This decrease was likely due to the already low incidence rates in Shanghai starting from 2006 (average incidence rate = 4.3/100,000 population/year during 2006–2007, the lowest among host PLADs; Fig 1c), which made transmission prone to stochastic fadeout. It was also likely due to limited susceptibility replenishment from connections to Jiangsu and Zhejiang, origin PLADs that had lower population susceptibilities (before the 2010 nationwide SIA, *S*_Jiangsu_% = 6.2%, *S*_Zhejiang_% = 6.0%, both lower than *S*_Shanghai_% = 6.3%; S8a Fig). Origin PLADs showed varying degrees of incidence reduction (S3 Table; see Hebei in Fig 5c as an example). Origin PLADs with larger migrant worker proportions had greater incidence reductions (Spearman’s rank correlation test, ρ = 0.85 (95% CI: (0.42, 0.97)), *P* = 3.1 × 10^-5^), with 7 of 16 origin PLADs (excluding Jiangsu and Zhejiang) showing significant reductions. PLADs that were neither hosts nor origins were minimally impacted (S3 Table; see Yunnan in Fig 5d as an example). Taken together, migrant workers might have facilitated the introduction of measles from their host PLADs to their origin PLADs.

Our calibrated model estimated higher population susceptibility in origin PLADs than that in host PLADs during 2005–2014 (before the 2010 nationwide SIA: Mann–Whitney *U* test, *r* = 0.71 (95% CI: (0.50, 0.82)), *P* = 3.8 × 10^-7^, average *S*_host_% = 6.2%, average *S*_origin_% = 7.4%, see *S*_preSIA_% across PLADs in S8a Fig; after the nationwide SIA: *r* = 0.71 (95% CI: (0.51, 0.83)), *P* = 3.8 × 10^-7^, average *S*_host_% = 6.1%, average *S*_origin_% = 7.0%, see *S*_postSIA_% across PLADs in S8b Fig). To test the impact of susceptibility replenishment from migrant workers, we conducted a third analysis assuming equal population susceptibility between incoming migrant workers and local populations in host PLADs (“matching population susceptibility” scenario). This scenario estimated a significant 35.8% national incidence reduction and significant reductions across host PLADs (Table 1). Shanghai showed a smaller reduction compared with other host PLADs, likely due to its connections with Jiangsu and Zhejiang, origin PLADs with lower population susceptibilities. The impact on host PLADs became evident one year into the simulation (see Jiangsu in Fig 5a and Beijing in Fig 5b as examples). This appeared as epidemic fadeouts from summer through early winter in 2005, with a subsequent 72.5% average incidence reduction compared with baseline before migrant workers began arriving in host PLADs. Similarly, the 2010 nationwide SIA, by reducing population susceptibility nationwide, led to large incidence reductions in 2011–2012, particularly in host PLADs (Fig 3e–3h). In origin PLADs, greater incidence reductions were associated with larger migrant worker proportions (Spearman’s rank correlation test, ρ = 0.64 (95% CI: (0.10, 0.87)), *P* = 0.008), with one origin PLAD showing significant reduction (S3 Table). PLADs that were neither hosts nor origins were minimally impacted (S3 Table). These results suggest that susceptibility replenishment from under-vaccinated migrant workers significantly contributed to measles outbreaks in host PLADs.

Lastly, in the fourth analysis, we tested a potential intervention of vaccinating migrant workers prior to their departure (“pre-migration vaccination” scenario with vaccination coverage ranging from 25% to 100%). Under 100% vaccination coverage, this scenario estimated a significant 50.9% national incidence reduction and significant reductions across host PLADs (Table 1). Similar to the two previous scenarios, origin PLADs with larger migrant worker proportions showed greater incidence reductions (Spearman’s rank correlation test, ρ = 0.95 (95% CI: (0.73, 0.99)), *P* = 2.7 × 10^-8^), although this effect was more pronounced, with 10 out of 16 origin PLADs showing significant reductions (S3 Table). As with the other scenarios, minimal impacts were seen in PLADs that were neither hosts nor origins (S3 Table). Additionally, vaccination coverage levels of 75%, 50%, and 25% estimated significant national incidence reductions of 47.2%, 43.0%, and 33.0%, respectively (S4 Table). These lower coverage scenarios showed similar patterns of reduction to the 100% coverage scenario across host, origin, and other PLADs.

## Discussion

In this study, we investigated the role of inter-PLAD rural-to-urban migrant workers in contributing to measles epidemics in China from 2005 to 2014. We developed a metapopulation SEIR model integrated with a migrant worker network and a traveler network to simulate measles transmission dynamics in both migrant worker and local subpopulations. Using this model, we identified key migrant worker subpopulations that contributed substantially to measles outbreaks in host PLADs despite sustained high vaccination coverage exceeding 95% in these host PLADs, and quantified the impacts of migrant worker mobility on measles epidemics through case importation and susceptibility replenishment.

The model identified key epidemiological connections between origin and host PLADs. In northern China, migrant workers from Hebei and Shandong were key contributors to outbreaks in Beijing and Tianjin. These PLADs were grouped together in the Infomap community #1 (Fig 1b) corresponding to the Beijing–Tianjin–Hebei Industrial Cluster. In southern China, the inferred connections were more complex. Anhui and Sichuan were identified as key origin PLADs contributing to outbreaks across multiple southern host PLADs. Specifically, migrant workers from Anhui contributed substantially to outbreaks in Jiangsu, Shanghai, and Zhejiang, corresponding to the Yangtze River Delta (Infomap community #3, Fig 1b), and those from Sichuan contributed substantially in all southern host PLADs. In contrast to other key epidemiological connections characterized by geographic contiguity, those originating from Sichuan represented long-distance epidemiological connections between the western origin PLAD and eastern host PLADs. While previous epidemiological studies typically reported aggregated incidence among migrant workers [9–11], our results provide detailed origin-host epidemiological connections that could support targeted vaccination strategies for measles control.

Counterfactual simulation removing traveler mobility (“no travelers” scenario) showed minimal impacts on measles epidemics (national incidence reduced by 0.3% compared with the baseline, Table 1). Given low measles incidence across PLADs during the study period (average annual incidence rate: 7.1/100,000 population/year from 2005 to 2010 before the nationwide SIA, and 1.8/100,000 population/year from 2011 to 2014), traveler-related seeding effect was likely limited. Additionally, because travelers typically remained in destination PLADs only briefly, their mobility had little influence on local population susceptibility. This finding contrasts with other respiratory infectious diseases, such as influenza [27–29] and SARS-CoV-2 [29,30], for which travelers or commuters facilitated broader geographic spread, potentially due to stronger seeding effect or higher population susceptibility.

Counterfactual simulation removing case importation through migrant worker mobility (“no case importation” scenario) resulted in a 15.4% reduction in national measles incidence (Table 1), primarily in origin PLADs. The magnitude of incidence reduction was positively correlated with the proportion of migrant workers in origin PLADs, suggesting that the strength of this seeding effect depended on the volume of migrant worker flows returning to their origin PLADs during the pre-CNY periods. These results emphasize the importance of controlling measles transmission in host PLADs, as endemic transmission therein could seed outbreaks in migrant worker origin PLADs. Effective control in host PLADs would therefore reduce measles incidence locally and in the connected origin PLADs.

Our model estimated higher population susceptibility in origin PLADs compared to host PLADs. To test the impact of susceptibility replenishment from migrant workers, we simulated a counterfactual scenario in which incoming migrant workers had susceptibility equal to that of local subpopulations in host PLADs (“matching population susceptibility” scenario). This scenario resulted in a 35.8% reduction in national incidence (Table 1), with significant reductions in host PLADs and varying reductions in origin PLADs. This result suggests that susceptibility replenishment from under-vaccinated migrant workers significantly contributed to measles outbreaks in host PLADs. Additionally, the reduced incidence in host PLADs led to further reductions in origin PLADs by weakening the seeding effect, and the degree of which was positively correlated with the proportion of migrant workers in origin PLADs.

The results from the counterfactual scenario, in which population susceptibility was matched between incoming migrant workers and local subpopulations in host PLADs, suggest that operational intervention strategies such as pre-migration vaccination could effectively control measles transmission in host PLADs and in turn reduce incidence in origin PLADs. Our counterfactual simulation of vaccinating migrant workers before their departure (“pre-migration vaccination” scenario), under coverage ranging from 25% to 100% and vaccine effectiveness of 95%, resulted in national incidence reductions from 33.0% to 50.9% (S4 Table). Significant reductions occurred in all host PLADs and in the majority of origin PLADs (origin PLADs excluding Jiangsu and Zhejiang), with larger reductions in origin PLADs having higher migrant worker proportions. Beijing has pioneered migrant worker-targeted SIAs since 2005 (e.g., 0.8 million vaccinated among 5.7 million migrant workers in 2009, i.e., 14.0% coverage) [31,32], which helped reduce, though not eliminate, measles transmission. Here, our results suggest that synchronized nationwide vaccination programs with higher coverage targeting migrant workers may more effectively control endemic transmission in host PLADs, origin PLADs, and throughout China. In practice, achieving high coverage in China may be constrained by limited awareness of vaccination history [33] and barriers to obtaining effective health insurance coverage and healthcare access for migrant workers (e.g., employer reluctance to provide insurance due to high premiums, and limited portability of rural household registration-linked insurance that may not cover outpatient services outside their home PLAD, which can reduce healthcare utilization [34]). Addressing these issues through improved vaccination outreach, as well as policies that improve effective insurance coverage and access to preventive services for migrant workers in host PLADs, may improve the feasibility of migrant worker-targeted vaccination programs.

Our study has several limitations. First, we only modeled measles transmission at the PLAD level, so we could not capture inhomogeneous mixing and migrant worker mobility at finer spatial resolutions. Thus, we were unable to simulate granular, localized outbreaks [14] and the irregular outbreaks observed in PLADs that were neither hosts nor origins. Second, we did not incorporate age structure, which may oversimplify age-dependent immunological profiles and contact patterns. However, given that migrant workers were predominantly working-age adults [18], routine childhood vaccination rate was high [35], and more limited epidemic impact of school-aged children during the study period [15], the resulting impact on our conclusions is likely limited. Third, although we constructed the traveler and migrant worker networks during 2005–2014 based on mobility patterns from 2015–2019 and annual flow volumes from official reports, flow variability could not be fully captured. Fourth, we attempted to model the mobility of transient migrant workers who failed to secure employment, in addition to long-term migrant workers, but we did not fully capture the magnitude and complexity of their mobility patterns due to limited data. Fifth, we did not incorporate unsynchronized PLAD-level SIAs and potential changes in case reporting due to a lack of relevant data, which may have partially contributed to worsened model performance particularly after 2009 (S1 Table). Despite these limitations, our study identified detailed epidemiological connections between PLADs and quantified the impact of inter-PLAD rural-to-urban migrant worker mobility on measles epidemics. In host PLADs with high routine childhood vaccination coverage, outbreaks persisted primarily due to susceptibility replenishment through inflows of under-vaccinated migrant workers. In origin PLADs, measles epidemics might have been synchronized and facilitated by case importation of exposed and infectious migrant workers returning from endemic host PLADs. These findings offer mechanistic insights into how migrant worker mobility shaped measles epidemic dynamics, which could inform public health interventions for measles control in China.

## Methods and materials

### Study data

Monthly measles incidence data for each PLAD from 2005 to 2014 were sourced from the Data-center of China Public Health Science [36]. City-level daily mobility data from February 3, 2015, to February 2, 2019, were sourced from Tencent mobile device location service (travel purposes unavailable). The dataset included 32.6 billion travel records from 363 of 368 cities at the prefecture, sub-provincial, and provincial levels in China. Demographic data, including birth and death rates, the sizes of total populations, and per-capita GRP were obtained from the National Bureau of Statistics of China [20]. Immunization rates were estimated as described previously [15]. Details on the estimations of inter-PLAD rural-to-urban migrant worker population sizes are provided in S1 Text “Estimation of inter-PLAD rural-to-urban migrant worker population sizes”.

### Construction of inter-PLAD traveler network and migrant worker network during 2005–2014

We constructed the inter-PLAD traveler network and migrant worker network for 2005–2014 based on the 2015–2019 mobility data. Because mobility data are not available for 2005–2014, we first examined mobility patterns using the 2015–2019 data. We observed consistent patterns within each year (volume surges during national holidays, while CNY showing surges in the pre- and post-holiday periods; S9 Fig). Therefore, we used mobility patterns from 2015–2019, as well as annual traveler volumes and migrant worker population sizes from 2005–2014 to reconstruct mobility networks during 2005–2014 with a two-step approach. This approach accounted for changing mobility patterns both within and between years during 2005–2014.

First, to capture the within-year mobility patterns, we aggregated the 2015–2019 daily flow volumes based on national holidays and inter-holiday periods. In the base mobility scenario, edge weights (mobility flow volumes) between the same node pairs (departure–arrival city pairs) were averaged for corresponding days within each holiday or inter-holiday period during 2015–2019. In addition, we constructed two alternative mobility scenarios for sensitivity analysis that introduce daily flow volume variability: 1) daily flow volumes were set to the minimum observed across 2015–2019 and 2) to the maximum observed. To match the spatial resolution of our analyses at the PLAD level, we further aggregated these city-level data by summing edge weights for the same departure–arrival PLAD pairs.

Second, to capture between-year mobility changes, we assigned the daily flow volumes from step 1 to corresponding days within each holiday or inter-holiday period during 2005–2014, and scaled them using the corresponding annual data (i.e., annual traveler volumes for the traveler network and annual migrant worker population sizes for the migrant worker network).

Specifically, to construct the traveler network representing general-purpose travel in step 2, we scaled the assigned daily flow volumes to match reported annual traveler volumes [20]. To ensure flow mass balance (i.e., balance between cumulative inflow and outflow volumes through each PLAD over the 10-year period), traveler volumes were adjusted using iterative proportional fitting [37]. The resulting adjustment factors ranged from 0.92 to 1.08.

To construct the migrant worker network representing rural-to-urban worker migration in step 2, we assumed that migrant worker mobility during 2005–2014 was concentrated within the migration periods (defined as six weeks before and after the CNY holidays). Because migration periods could span multiple holidays and inter-holiday periods, and the exact overlap varied by year due to the shifting dates of the CNY based on the lunar calendar, we estimated migrant worker flows based on the constructed traveler flows. Specifically, for each year, we first estimated the total volume of migrant workers returning to their origin PLADs during the pre-CNY period, as well as the total volumes of recurring and new migrant workers moving to host PLADs during the post-CNY period. These estimates, which captured between-year variations associated with social and economic transformation, were calculated based on migrant worker population sizes for that year (see details in S1 Text “Estimation of inter-PLAD rural-to-urban migrant worker population sizes”), the proportion of migrant workers remaining in host PLADs during CNY [38], the average duration of stay as migrant workers [39], and the employment rate of migrant workers [40] (see formulas in S19–S22 Eq). Then, to estimate daily migrant worker volumes, the total migrant worker volumes were proportionally distributed based on the daily traveler volumes during the migration periods.

### Networked metapopulation SEIR model

The networked metapopulation SEIR model divided each calendar year into four periods based on migrant worker mobility patterns related to CNY: regular (no migration), pre-CNY (defined as six weeks before the CNY holidays; migrant workers return from host PLADs to origin PLADs), CNY holidays (one week; no migration), and post-CNY (six weeks after the CNY holidays; migrant workers move from origin PLADs to host PLADs) periods. PLADs ranked highest in both the proportion of migrant workers (from 35.2% in Shanghai to 8.6% in Jiangsu) and per-capita GRP were determined as host PLADs in the model (seven host PLADs in total; S2 Fig). The next-highest PLADs by migrant worker proportion were Xinjiang (5.6%) and Tibet (4.2%), but their per-capita GRP ranked 17th and 28th, respectively, indicating that migration to these PLADs was less likely to represent rural-to-urban migration. For each host PLAD, all other PLADs were ranked based on their proportions of the total migrant worker population, and the top-ranked PLADs collectively accounting for >75% of the total migrant worker population were determined as the origin PLADs for that host PLAD (18 origin PLADs in total; S2 Fig). Jiangsu and Zhejiang served as both host and origin PLADs. The remaining eight PLADs were neither host nor origin PLADs. PLADs were further divided into subpopulations based on their host and origin roles. Each host PLAD was divided into a local subpopulation (including local residents and white-collar migrant workers with comparable population susceptibility) and migrant worker subpopulations (specifically rural-to-urban migrant workers) from corresponding origin PLADs (e.g., Beijing, Fig 3b). Each origin PLAD was divided into a local subpopulation and returned migrant worker subpopulations (specifically rural-to-urban migrant workers) from corresponding host PLADs (e.g., Hebei, Fig 3c). PLADs that served as both host and origin contained all three types of subpopulations (e.g., Jiangsu, Fig 3a), and PLADs that were neither hosts nor origins contained only local subpopulations (e.g., Yunnan, Fig 3d).

The model framework consisted of a basic SEIR model for each local, migrant worker, and returned migrant worker subpopulation in each PLAD. Subpopulations across PLADs were interconnected through a migrant worker network and a traveler network. Each basic SEIR model included transmission terms determined by population contact patterns and climate forcing (accounting for the effects of humidity and temperature on measles transmission), demographic processes (births and deaths), and routine childhood vaccination. Model formulation details are provided in S1 Text “Networked metapopulation SEIR model”. Additionally, the nationwide SIA conducted in 2010 was modeled, and the details of the SIA modeling are provided in S1 Text “Modeling nationwide SIA”.

The model was initialized with 2 million realizations generated using initialization methods for model state variables (Susceptible population informed by a population model (see S10 Fig for prior ranges and posterior distributions after calibration); Exposed and Infectious populations informed by incidence rates reported for the first week of 2005) and Latin Hypercube sampling of model parameters (reporting rate informed by a time series susceptible–infected–recovered model during 2005–2008 [8,15,41]; other parameters informed by estimates from the literature [15]). Model initialization details are provided in S1 Text “Model initialization”. To calibrate the model, we first calculated the likelihood for each trajectory by comparing simulated incidence with observed incidence time series from 2005 to 2008 (a period with regular annual outbreaks), assuming a normal distribution $N(z_{t},{\left(\frac{\frac{\text{1}}{n_{t}}\sum_{k=t-\text{2}}^{t}z_{k}}{\text{2}}\right)}^{\text{2}}+{\text{1}\text{0}}^{\text{4}})$, where $z_{t}$ is the observed incidence at time t, and $n_{t}$ is the number of available observations in the window [t−2,t]. We also filtered out trajectories whose proportions of incidence attributed to migrant worker subpopulations in host PLADs fell outside ranges reported in the literature [9,11,42–47]. We then selected the 200 trajectories (i.e., the top 0.01%) with the highest likelihoods and resampled from these trajectories with replacement, using probability proportional to their likelihoods, to form a calibrated ensemble of 3,000 trajectories. The median estimates of calibrated model parameters are shown in S11 and S12 Figs. To simulate epidemic dynamics over the entire study period from 2005 to 2014 and to account for model stochasticity, each trajectory was reinitialized and rerun 10 times for this period. The resulting model ensemble served as the baseline for downstream analyses.

### Construction of counterfactual scenarios

Four counterfactual scenarios were constructed based on the networked metapopulation SEIR model. 1) No travelers: The traveler volumes in the traveler network were set to 0; 2) No case importation: For each migrant worker flow, the volumes of exposed and infectious individuals were set to 0, and the same total migrant worker flow volume was distributed between susceptible and recovered/immunized individuals in proportion to their original ratio; 3) Matching population susceptibility: For each migrant worker flow into a host PLAD, the population susceptibility of migrant workers was set to mirror that of the local subpopulation in the receiving host PLAD, the volumes of exposed and infectious individuals were kept unchanged, and the volume of recovered/immunized individuals was adjusted to preserve the total migrant worker flow volume; 4) Pre-migration vaccination: We assumed that migrant workers were vaccinated prior to departure to host PLADs with 100%, 75%, 50%, and 25% coverage and 95% effectiveness. For each migrant worker flow into a host PLAD, vaccination was implemented by transitioning susceptible individuals to the recovered/immunized compartment to achieve a population susceptibility that was a weighted average of 5% among vaccinated migrant workers and the original susceptibility among unvaccinated migrant workers, and the volumes of exposed and infectious individuals were kept unchanged.

## Supporting information

S1 Fig Relative weighted out-degree of the inter-PLAD traveler network from 2005 to 2014. (DOCX)

S2 Fig Population compositions of PLADs hosting the largest proportions of inter-PLAD rural-to-urban migrant workers (host PLADs). For each host PLAD, individual sources of migrant workers (origin PLADs) are ordered by their proportions from the smallest at the top to the largest at the bottom. They collectively account for >75% of the total migrant worker population.
(DOCX)

S3 Fig Simulated measles incidence from the calibrated model ensemble for host PLADs, compared with observed incidence (crosses). (DOCX)

S4 Fig Simulated measles incidence from the calibrated model ensemble for origin PLADs, compared with observed incidence (crosses); part 1. (DOCX)

S5 Fig Simulated measles incidence from the calibrated model ensemble for origin PLADs, compared with observed incidence (crosses); part 2. (DOCX)

S6 Fig Simulated measles incidence from the calibrated model ensemble for PLADs neither hosts nor origins, compared with observed incidence (crosses). (DOCX)

S7 Fig Key epidemiological connections among PLADs under (a) the alternative mobility scenario in which daily mobility flow volumes for each corresponding day within each national holiday or inter-holiday period were set to the minimum observed in the original 2015–2019 mobility data, and (b) the alternative mobility scenario in which the flow volumes were set to the maximum observed. PLAD and country boundary basemaps were obtained from the publicly available Natural Earth shapefiles (https://www.naturalearthdata.com/downloads/; terms of use: https://www.naturalearthdata.com/about/terms-of-use/), via the R packages rnaturalearth (v1.0.1) [24] and sf (v1.0-15) [25].
(DOCX)

S8 Fig Median estimates of population susceptibilities (a) before and (b) after the nationwide SIA in 2010. PLAD and country boundary basemaps were obtained from the publicly available Natural Earth shapefiles (https://www.naturalearthdata.com/downloads/; terms of use: https://www.naturalearthdata.com/about/terms-of-use/), via the R packages rnaturalearth (v1.0.1) [24] and sf (v1.0-15) [25].
(DOCX)

S9 Fig Inter-PLAD mobility flow volumes aggregated by national holidays and inter-holiday periods from the original 2015–2019 mobility data. National holidays include New Year (“ny” on the x-axis), Chinese New Year (“cny”), Qingming Festival (“qingming”), Labor Day (“labor”), Dragon Boat Festival (“dragonboat”), Mid-Autumn Festival (“midautumn”), and National Day (“national”). “holiday_pre” and “holiday_post” indicate the periods one week before and after the holiday (three weeks for Chinese New Year), respectively. “holiday A_holiday B” indicate the inter-holiday period between the two holidays.
(DOCX)

S10 Fig Prior ranges and posterior distributions of initial population susceptibility by PLAD. For each PLAD, short vertical lines indicate the prior range estimated by the population model, and the density plot indicates the posterior distribution after calibration.
(DOCX)

S11 FigMedian estimates of calibrated model parameters, part 1.Bolded fonts indicate host PLADs. $R_{\text{0},cont}$: basic reproductive number ($R_{\text{0}}$) based on contact; $R_{\text{0},min,clim}$: minimum $R_{\text{0}}$ in an absolute humidity and temperature-forced model; $R_{\text{0},diff,clim}$: difference between maximum and minimum $R_{\text{0}}$ in an absolute humidity and temperature-forced model; $\overset{\prime }{\underset{\text{2}}{\beta }}$: transmission rate between subpopulations originally from different PLADs ($\beta_{\text{2}}$), relative to the transmission rate within each subpopulation ($\beta_{\text{1}}$); $\overset{\prime }{\underset{\text{3}}{\beta }}$: transmission rate between subpopulations originally from a same PLAD ($\beta_{\text{3}}$), relative to $\beta_{\text{1}}$; $m_{\text{1}}$: mixing exponent within subpopulation. PLAD and country boundary basemaps were obtained from the publicly available Natural Earth shapefiles (https://www.naturalearthdata.com/downloads/; terms of use: https://www.naturalearthdata.com/about/terms-of-use/), via the R packages rnaturalearth (v1.0.1) [24] and sf (v1.0-15) [25].(DOCX)

S12 FigMedian estimates of calibrated model parameters, part 2.$m_{\text{2}}$: mixing exponent between subpopulations originally from different PLADs; $m_{\text{3}}$: mixing exponent between subpopulations originally from a same PLAD; Z: latent period; D: infectious period; ρ: reporting rate. PLAD and country boundary basemaps were obtained from the publicly available Natural Earth shapefiles (https://www.naturalearthdata.com/downloads/; terms of use: https://www.naturalearthdata.com/about/terms-of-use/), via the R packages rnaturalearth (v1.0.1) [24] and sf (v1.0-15) [25].(DOCX)

S13 FigSchematic of the study time periods and migrant worker flows.**(a)** Schematic of the defined time periods and their durations based on the migrant worker mobility patterns related to CNY in a typical year. $t_{\text{1}}$: regular period (no migration); $t_{\text{2}}$: pre-CNY migration period (duration $T_{preCNY}$: 6 weeks before the CNY’s eve $t_{CNYE}$); $t_{\text{3}}$: CNY period (duration $T_{CNY}$: 1 week; no migration); $t_{\text{4}}$: post-CNY migration period (duration $T_{postCNY}$: 6 weeks); $\overset{\prime }{\underset{\text{1}}{t}}:\;$period during which migrant workers who failed to secure employment return (duration of job seeking $T_{job\;seek}$: 8 weeks). Schematic of migrant worker flows between a focal PLAD i serving as both a host and an origin PLAD (indicated by a larger pie), and connected PLADs j and k (indicated by smaller pies), during **(b)** $t_{\text{1}}$, **(c)** $t_{\text{2}}$, **(d)** $t_{\text{3}}$, **(e)** $t_{\text{4}}$, and **(f)** $\overset{\prime }{\underset{\text{1}}{t}}$. For a focal host PLAD i, the migrant worker flows enclosed by green lines apply, and the returned migrant worker subpopulation $N_{k,i,r}$ should be ignored. For a focal origin PLAD i, the migrant worker flows enclosed by blue lines apply, and the migrant worker subpopulation $N_{i,j}$ should be ignored. A focal PLAD i that is neither host nor origin contains only a local subpopulation and has no migrant worker flows. Model details of migrant worker flows are provided in S19–22 Eqs.(DOCX)

S1 Table Summary statistics of model fits for 2005–2008 and out-of-sample validation for January 2009–September 2010 (prior to the nationwide SIA). RMSE: Root Mean Square Error; RRMSE: Relative Root Mean Square Error; *r*: correlation coefficient; peak time difference: mean of absolute differences (in months) between observed and simulated peak timings.
(DOCX)

S2 Table Mobility sensitivity analysis comparing the two alternative mobility scenarios with the base scenario. Under alternative scenario #1, daily mobility flow volumes for each corresponding day within each national holiday or inter-holiday period were set to the minimum observed in the original 2015–2019 mobility data; under alternative scenario #2, the flow volumes were set to the maximum observed. *r*: correlation coefficient between the simulated incidence time series under an alternative scenario and the base scenario; incidence difference: relative difference in cumulative incidence over 2005–2014 for an alternative scenario compared with the base scenario.
(DOCX)

S3 Table Relative differences in cumulative incidence between the four counterfactual scenarios and the baseline in origin PLADs and in PLADs that were neither host nor origin. (DOCX)

S4 Table Relative differences in cumulative incidence between the “pre-migration vaccination” scenario at different coverage levels and the baseline in China and individual PLADs. (DOCX)

S5 Table Values of constant model parameters. (DOCX)

S6 Table Initial ranges of model state variables and parameters. (DOCX)

S1 Text Supplementary Text. (DOCX)
