# Supplementary figures and images for: Rural-to-urban migrant worker mobility shaped measles epidemics in China
Source: PLoS Comput Biol. 2026 Apr 10;22(4):e1014182. doi: 10.1371/journal.pcbi.1014182 (PMC13170960; doi:10.1371/journal.pcbi.1014182)

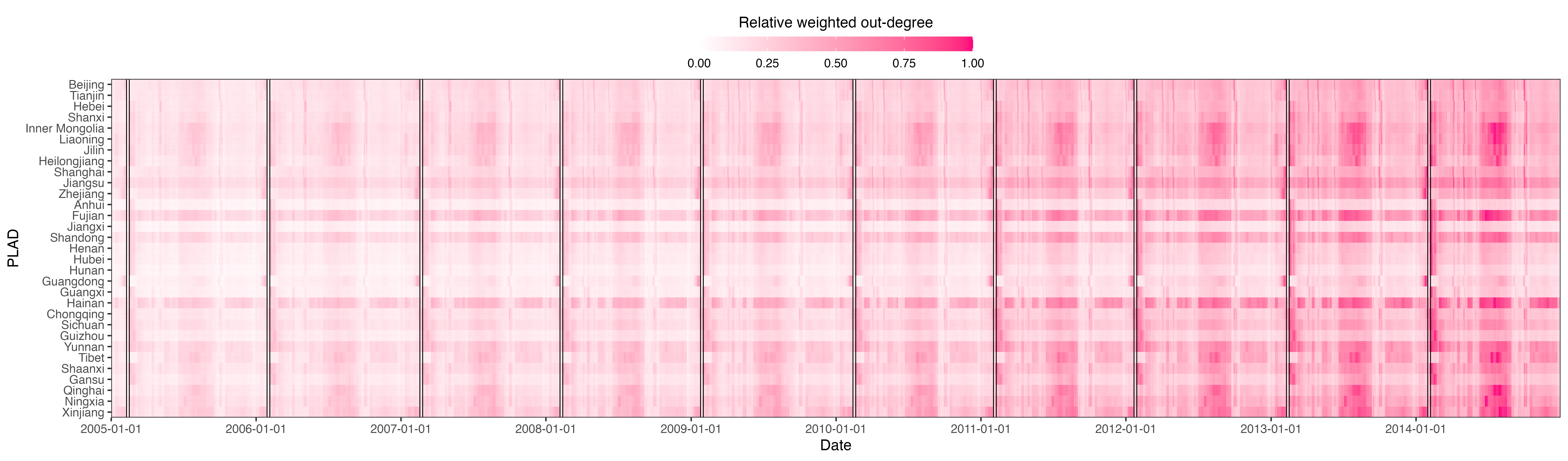


**S1 Fig.** Relative weighted out-degree of the inter-PLAD traveler network from 2005 to 2014.

Supplement: S1 Fig — (DOCX) [file pcbi.1014182.s001.docx]
