# Supplementary material for: Rural-to-urban migrant worker mobility shaped measles epidemics in China
Source: PLoS Comput Biol. 2026 Apr 10;22(4):e1014182. doi: 10.1371/journal.pcbi.1014182 (PMC13170960; doi:10.1371/journal.pcbi.1014182)
Supplement: S2 Fig — For each host PLAD, individual sources of migrant workers (origin PLADs) are ordered by their proportions from the smallest at the top to the largest at the bottom. They collectively account for >75% of the total migrant worker population. (DOCX) [file pcbi.1014182.s002.docx]

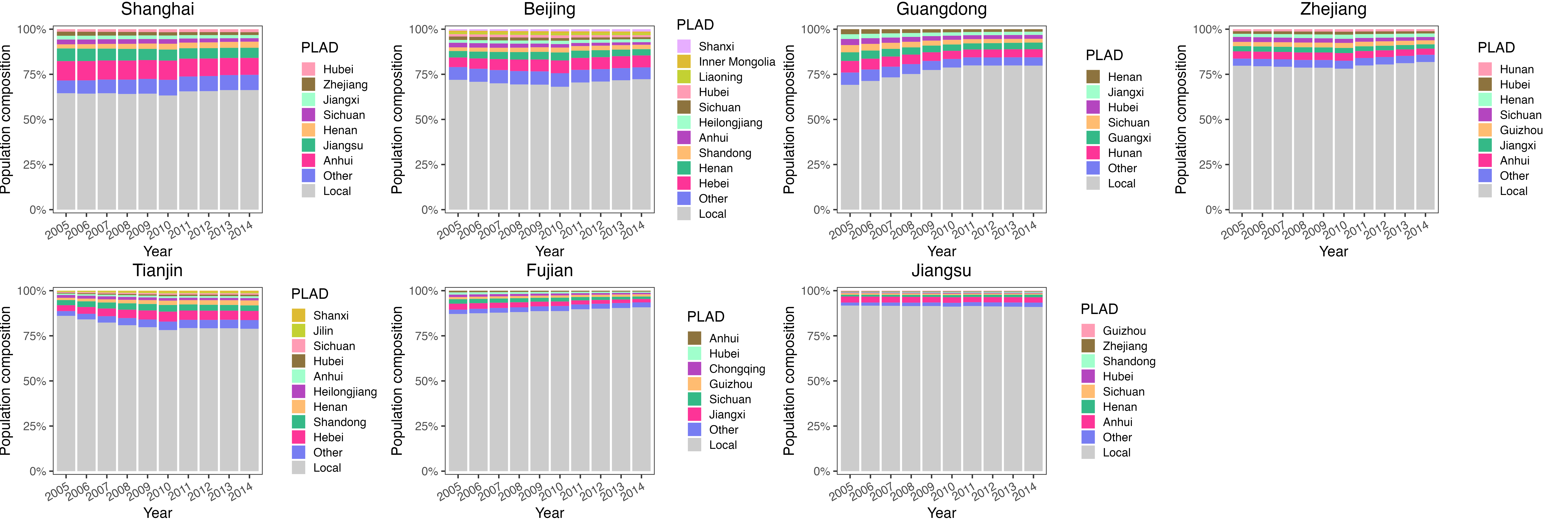


**S2 Fig.** Population compositions of PLADs hosting the largest proportions of inter-PLAD rural-to-urban migrant workers (host PLADs). For each host PLAD, individual sources of migrant workers (origin PLADs) are ordered by their proportions from the smallest at the top to the largest at the bottom. They collectively account for >75% of the total migrant worker population.
