# Supplementary material for: Rural-to-urban migrant worker mobility shaped measles epidemics in China
Source: PLoS Comput Biol. 2026 Apr 10;22(4):e1014182. doi: 10.1371/journal.pcbi.1014182 (PMC13170960; doi:10.1371/journal.pcbi.1014182)
Supplement: S3 Fig — (DOCX) [file pcbi.1014182.s003.docx]

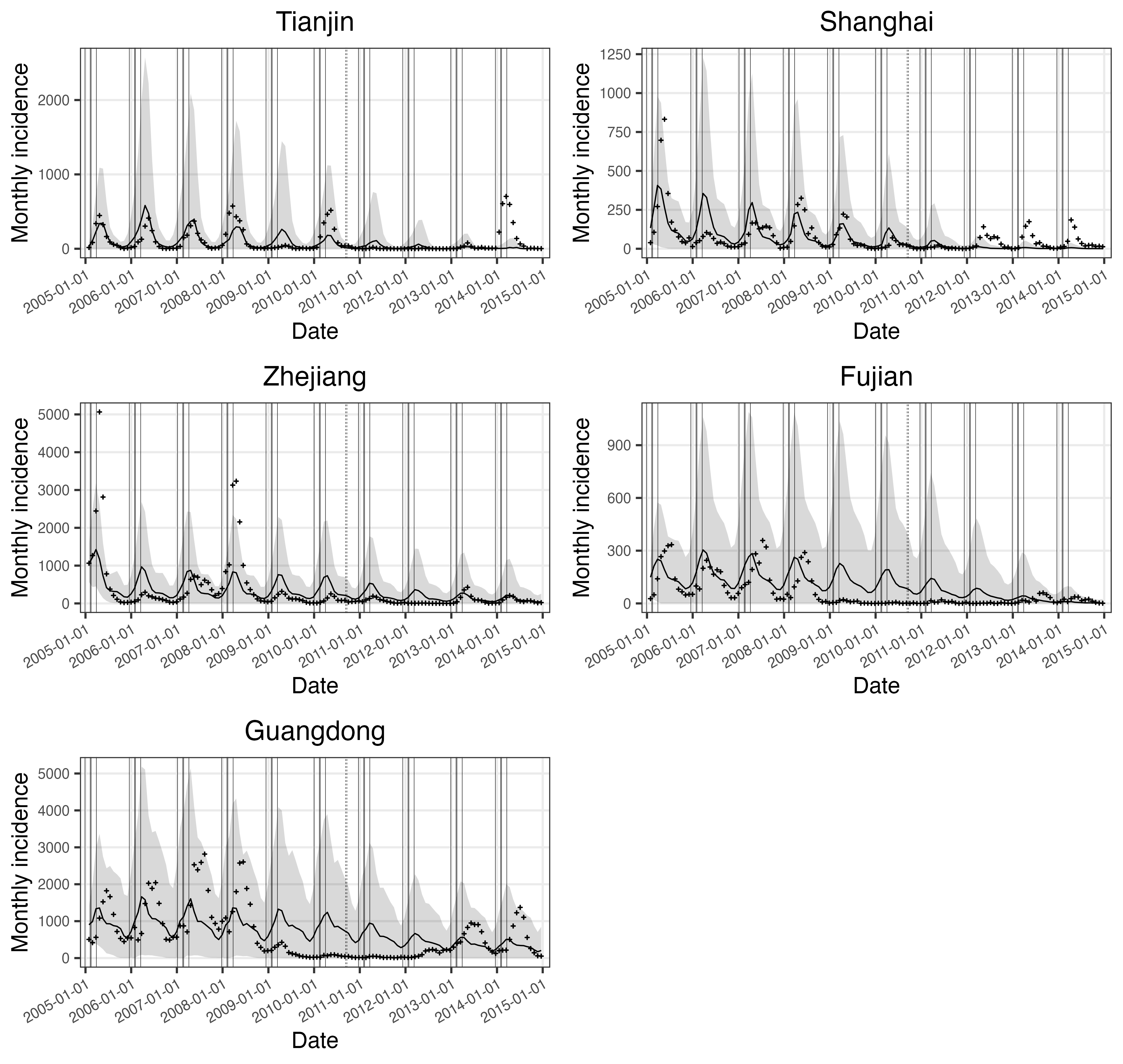


**S3 Fig.** Simulated measles incidence from the calibrated model ensemble for host PLADs, compared with observed incidence (crosses).
