# Supplementary material for: Rural-to-urban migrant worker mobility shaped measles epidemics in China
Source: PLoS Comput Biol. 2026 Apr 10;22(4):e1014182. doi: 10.1371/journal.pcbi.1014182 (PMC13170960; doi:10.1371/journal.pcbi.1014182)
Supplement: S7 Fig — PLAD and country boundary basemaps were obtained from the publicly available Natural Earth shapefiles (https://www.naturalearthdata.com/downloads/; terms of use: https://www.naturalearthdata.com/about/terms-of-use/), via the R packages rnaturalearth (v1.0.1) [24] and sf (v1.0-15) [25]. (DOCX) [file pcbi.1014182.s007.docx]

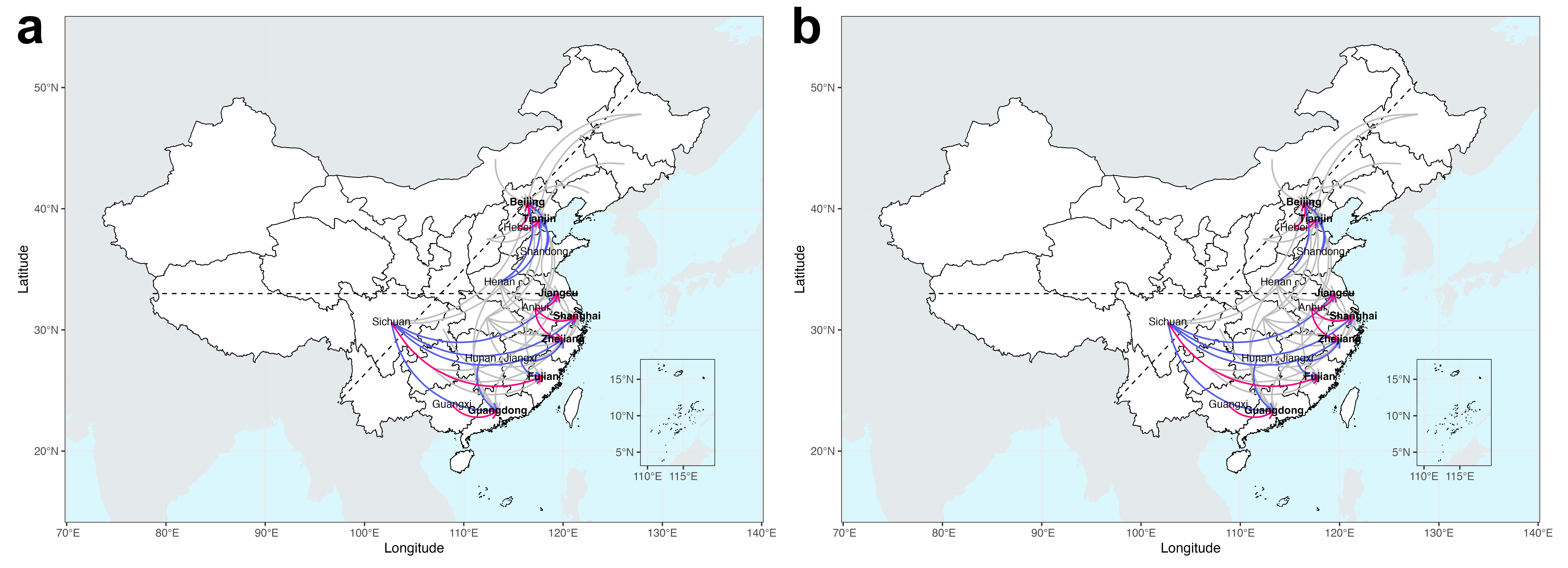


**S7 Fig.** Key epidemiological connections among PLADs under (**a**) the alternative mobility scenario in which daily mobility flow volumes for each corresponding day within each national holiday or inter-holiday period were set to the minimum observed in the original 2015–2019 mobility data, and (**b**) the alternative mobility scenario in which the flow volumes were set to the maximum observed. PLAD and country boundary basemaps were obtained from the publicly available Natural Earth shapefiles (<https://www.naturalearthdata.com/downloads/>; terms of use: <https://www.naturalearthdata.com/about/terms-of-use/>), via the R packages rnaturalearth (v1.0.1) (1) and sf (v1.0-15) (2).

**References**

1. Massicotte P, South A. rnaturalearth: World map data from natural earth. 2025.

2. Pebesma E, Bivand R. Spatial data science: With applications in R. New York: Chapman and Hall/CRC; 2023.
