# Supplementary material for: Rural-to-urban migrant worker mobility shaped measles epidemics in China
Source: PLoS Comput Biol. 2026 Apr 10;22(4):e1014182. doi: 10.1371/journal.pcbi.1014182 (PMC13170960; doi:10.1371/journal.pcbi.1014182)
Supplement: S9 Fig — National holidays include New Year (“ny” on the x-axis), Chinese New Year (“cny”), Qingming Festival (“qingming”), Labor Day (“labor”), Dragon Boat Festival (“dragonboat”), Mid-Autumn Festival (“midautumn”), and National Day (“national”). “holiday_pre” and “holiday_post” indicate the periods one week before and after the holiday (three weeks for Chinese New Year), respectively. “holiday A_holiday B” indicate the inter-holiday period between the two holidays. (DOCX) [file pcbi.1014182.s009.docx]

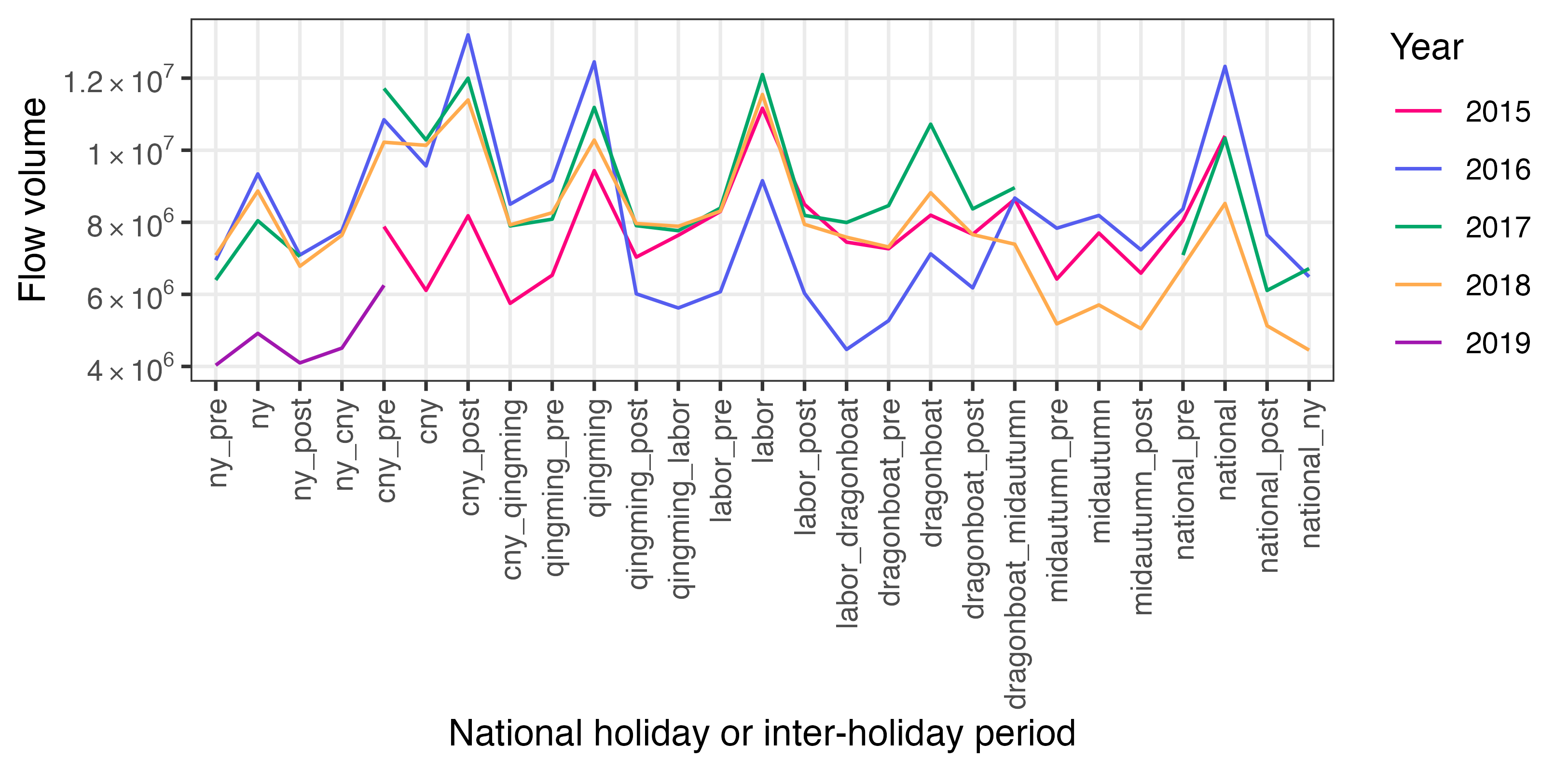


**S9 Fig.** Inter-PLAD mobility flow volumes aggregated by national holidays and inter-holiday periods from the original 2015–2019 mobility data. National holidays include New Year (“ny” on the x-axis), Chinese New Year (“cny”), Qingming Festival (“qingming”), Labor Day (“labor”), Dragon Boat Festival (“dragonboat”), Mid-Autumn Festival (“midautumn”), and National Day (“national”). “holiday_pre” and “holiday_post” indicate the periods one week before and after the holiday (three weeks for Chinese New Year), respectively. “holiday A_holiday B” indicates the inter-holiday period between the two holidays.
