# Supplementary material for: Rural-to-urban migrant worker mobility shaped measles epidemics in China
Source: PLoS Comput Biol. 2026 Apr 10;22(4):e1014182. doi: 10.1371/journal.pcbi.1014182 (PMC13170960; doi:10.1371/journal.pcbi.1014182)
Supplement: S10 Fig — For each PLAD, short vertical lines indicate the prior range estimated by the population model, and the density plot indicates the posterior distribution after calibration. (DOCX) [file pcbi.1014182.s010.docx]

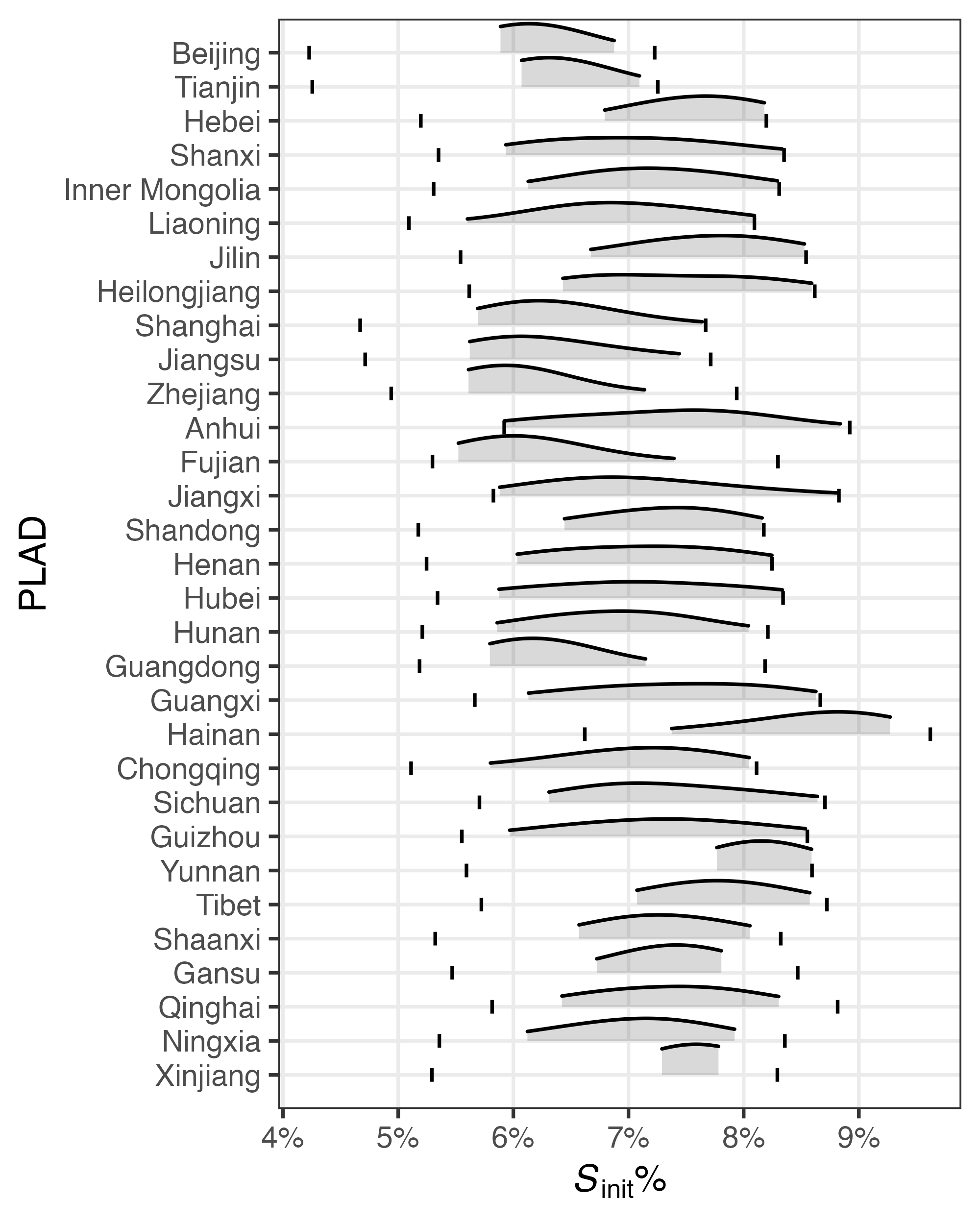


**S10 Fig.** Prior ranges and posterior distributions of initial population susceptibility by PLAD. For each PLAD, short vertical lines indicate the prior range estimated by the population model, and the density plot indicates the posterior distribution after calibration.
