# Supplementary material for: Rural-to-urban migrant worker mobility shaped measles epidemics in China
Source: PLoS Comput Biol. 2026 Apr 10;22(4):e1014182. doi: 10.1371/journal.pcbi.1014182 (PMC13170960; doi:10.1371/journal.pcbi.1014182)
Supplement: S11 Fig — Bolded fonts indicate host PLADs. R0,cont: basic reproductive number (R0) based on contact; R0,min,clim: minimum R0 in an absolute humidity and temperature-forced model; R0,diff,clim: difference between maximum and minimum R0 in an absolute humidity and temperature-forced model; β2′: transmission rate between subpopulations originally from different PLADs (β2), relative to the transmission rate within each subpopulation (β1); β3′: transmission rate between subpopulations originally from a same PLAD (β3), relative to β1; m1: mixing exponent within subpopulation. PLAD and country boundary basemaps were obtained from the publicly available Natural Earth shapefiles (https://www.naturalearthdata.com/downloads/; terms of use: https://www.naturalearthdata.com/about/terms-of-use/), via the R packages rnaturalearth (v1.0.1) [24] and sf (v1.0-15) [25]. (DOCX) [file pcbi.1014182.s011.docx]

**S11 Fig.** Median estimates of calibrated model parameters, part 1. Bolded fonts indicate host PLADs. $R_{0,cont}$: basic reproductive number ($R_{0}$) based on contact; $R_{0,min,clim}$: minimum $R_{0}$ in an absolute humidity and temperature-forced model; $R_{0,diff,clim}$: difference between maximum and minimum $R_{0}$ in an absolute humidity and temperature-forced model; $\beta_{2}^{'}$: transmission rate between subpopulations originally from different PLADs ($\beta_{2}$), relative to the transmission rate within each subpopulation ($\beta_{1}$); $\beta_{3}^{'}$: transmission rate between subpopulations originally from a same PLAD ($\beta_{3}$), relative to $\beta_{1}$; $m_{1}$: mixing exponent within subpopulation. PLAD and country boundary basemaps were obtained from the publicly available Natural Earth shapefiles (<https://www.naturalearthdata.com/downloads/>; terms of use: <https://www.naturalearthdata.com/about/terms-of-use/>), via the R packages rnaturalearth (v1.0.1) (1) and sf (v1.0-15) (2).

**References**

1. Massicotte P, South A. rnaturalearth: World map data from natural earth. 2025.

2. Pebesma E, Bivand R. Spatial data science: With applications in R. New York: Chapman and Hall/CRC; 2023.
