# Supplementary material for: Rural-to-urban migrant worker mobility shaped measles epidemics in China
Source: PLoS Comput Biol. 2026 Apr 10;22(4):e1014182. doi: 10.1371/journal.pcbi.1014182 (PMC13170960; doi:10.1371/journal.pcbi.1014182)
Supplement: S13 Fig — (a) Schematic of the defined time periods and their durations based on the migrant worker mobility patterns related to CNY in a typical year. t1: regular period (no migration); t2: pre-CNY migration period (duration TpreCNY: 6 weeks before the CNY’s eve tCNYE); t3: CNY period (duration TCNY: 1 week; no migration); t4: post-CNY migration period (duration TpostCNY: 6 weeks); t1′: period during which migrant workers who failed to secure employment return (duration of job seeking Tjob seek: 8 weeks). Schematic of migrant worker flows between a focal PLAD i serving as both a host and an origin PLAD (indicated by a larger pie), and connected PLADs j and k (indicated by smaller pies), during (b) t1, (c) t2, (d) t3, (e) t4, and (f) t1′. For a focal host PLAD i, the migrant worker flows enclosed by green lines apply, and the returned migrant worker subpopulation Nk,i,r should be ignored. For a focal origin PLAD i, the migrant worker flows enclosed by blue lines apply, and the migrant worker subpopulation Ni,j should be ignored. A focal PLAD i that is neither host nor origin contains only a local subpopulation and has no migrant worker flows. Model details of migrant worker flows are provided in S19–22 Eqs. (DOCX) [file pcbi.1014182.s013.docx]

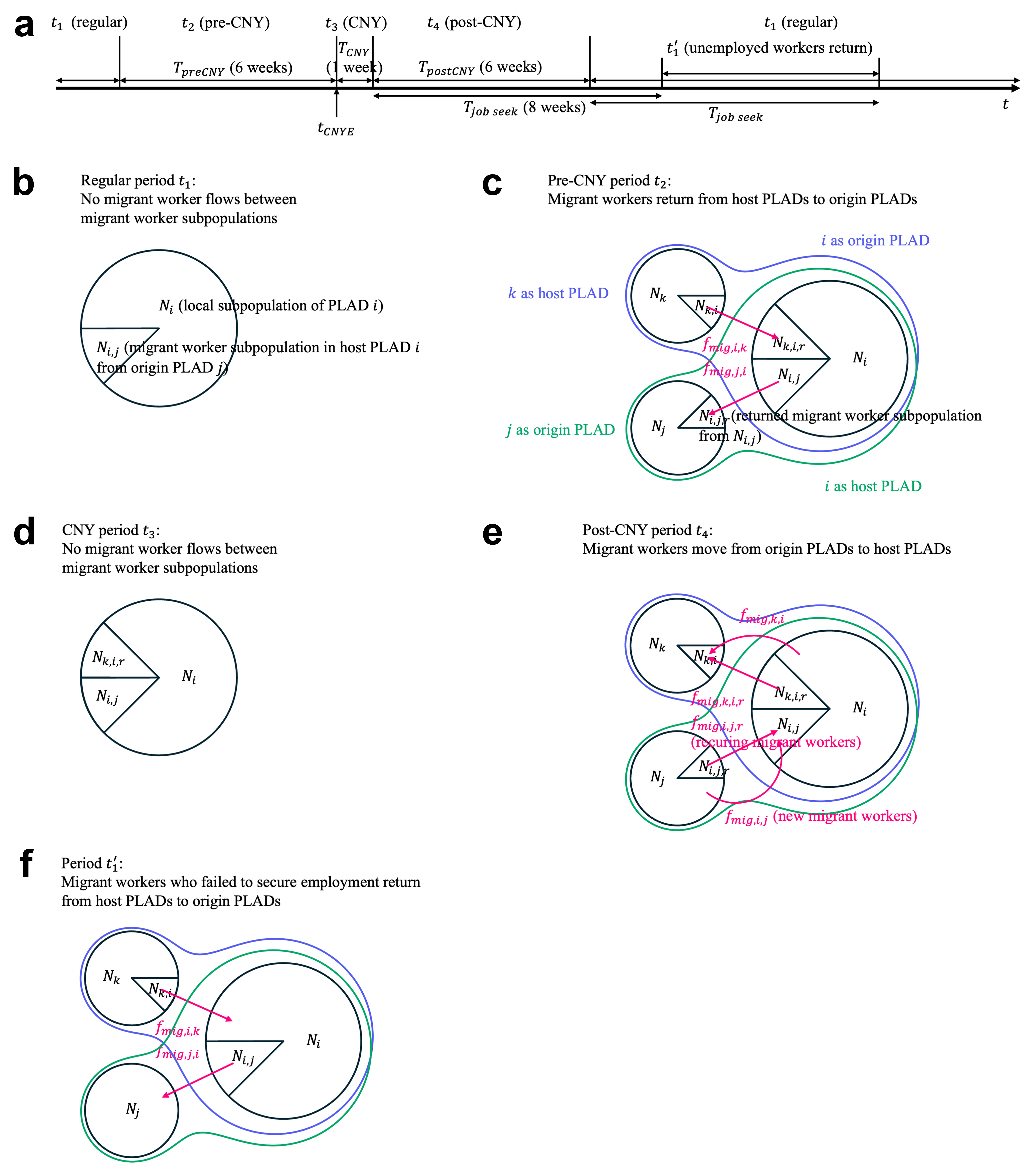


**S13 Fig.** (**a**) Schematic of the defined time periods and their durations based on the migrant worker mobility patterns related to CNY in a typical year. $t_{1}$: regular period (no migration); $t_{2}$: pre-CNY migration period (duration $T_{preCNY}$: 6 weeks before the CNY’s eve $t_{CNYE}$); $t_{3}$: CNY period (duration $T_{CNY}$: 1 week; no migration); $t_{4}$: post-CNY migration period (duration $T_{postCNY}$: 6 weeks); $t_{1}^{'}:$period during which migrant workers who failed to secure employment return (duration of job seeking $T_{job seek}$: 8 weeks). Schematic of migrant worker flows between a focal PLAD $i$ serving as both a host and an origin PLAD (indicated by a larger pie), and connected PLADs $j$ and $k$ (indicated by smaller pies), during (**b**) $t_{1}$, (**c**) $t_{2}$, (**d**) $t_{3}$, (**e**) $t_{4}$, and (**f**) $t_{1}^{'}$. For a focal host PLAD $i$, the migrant worker flows enclosed by green lines apply, and the returned migrant worker subpopulation $N_{k,i,r}$ should be ignored. For a focal origin PLAD $i$, the migrant worker flows enclosed by blue lines apply, and the migrant worker subpopulation $N_{i,j}$ should be ignored. A focal PLAD $i$ that is neither host nor origin contains only a local subpopulation and has no migrant worker flows. Model details of migrant worker flows are provided in S19–S22 Eq.
