# Supplementary material for: Rural-to-urban migrant worker mobility shaped measles epidemics in China
Source: PLoS Comput Biol. 2026 Apr 10;22(4):e1014182. doi: 10.1371/journal.pcbi.1014182 (PMC13170960; doi:10.1371/journal.pcbi.1014182)
Supplement: S1 Table — RMSE: Root Mean Square Error; RRMSE: Relative Root Mean Square Error; r: correlation coefficient; peak time difference: mean of absolute differences (in months) between observed and simulated peak timings. (DOCX) [file pcbi.1014182.s014.docx]

**S1 Table.** Summary statistics of model fits for 2005–2008 and out-of-sample validation for January 2009–September 2010 (prior to the nationwide SIA). RMSE: Root Mean Square Error; RRMSE: Relative Root Mean Square Error; *r*: correlation coefficient; peak time difference: mean of absolute differences (in months) between observed and simulated peak timings.

| PLAD type | PLAD | RMSE | | RRMSE | | *r* | | Peak time difference | |
| --- | --- | --- | --- | --- | --- | --- | --- | --- | --- |
|  |  | Fit | Validation | Fit | Validation | Fit | Validation | Fit | Validation |
| Host | Beijing | 85.7 | 136.3 | 0.397 | 0.847 | 0.935 | 0.650 | 1.00 | 0.50 |
|  | Tianjin | 102.9 | 140.7 | 0.769 | 1.462 | 0.761 | 0.396 | 0.75 | 1.00 |
|  | Shanghai | 127.9 | 40.4 | 1.101 | 0.821 | 0.570 | 0.769 | 1.25 | 0.50 |
|  | Jiangsu | 336.1 | 292.8 | 0.728 | 1.101 | 0.725 | 0.738 | 0.25 | 0.50 |
|  | Zhejiang | 858.7 | 304.8 | 1.308 | 2.642 | 0.641 | 0.667 | 1.00 | 1.00 |
|  | Fujian | 98.6 | 133.1 | 0.753 | 23.996 | 0.414 | 0.593 | 2.75 | 1.00 |
|  | Guangdong | 709.0 | 780.4 | 0.608 | 6.373 | 0.338 | 0.646 | 3.25 | 1.00 |
| Origin | Hebei | 439.6 | 912.5 | 1.137 | 1.130 | 0.874 | 0.686 | 0.25 | 0.50 |
|  | Shanxi | 274.7 | 137.9 | 0.812 | 4.669 | 0.797 | 0.821 | 0.50 | 0.50 |
|  | Inner Mongolia | 137.8 | 172.0 | 0.667 | 1.792 | 0.831 | 0.834 | 0.75 | 1.00 |
|  | Liaoning | 249.8 | 177.6 | 1.314 | 1.112 | 0.623 | 0.662 | 0.50 | 0.50 |
|  | Jilin | 291.7 | 239.0 | 1.980 | 1.964 | 0.508 | 0.630 | 0.50 | 0.50 |
|  | Heilongjiang | 201.4 | 624.2 | 1.821 | 1.927 | 0.593 | 0.630 | 0.50 | 0.50 |
|  | Anhui | 356.9 | 292.3 | 1.204 | 1.397 | 0.455 | 0.521 | 1.00 | 1.00 |
|  | Jiangxi | 117.0 | 145.0 | 0.760 | 2.073 | 0.634 | 0.359 | 1.75 | 1.50 |
|  | Shandong | 335.2 | 265.5 | 0.938 | 1.506 | 0.637 | 0.754 | 0.50 | 1.50 |
|  | Henan | 361.9 | 450.4 | 0.543 | 0.871 | 0.777 | 0.672 | 1.00 | 0.50 |
|  | Hubei | 232.9 | 261.2 | 0.820 | 1.274 | 0.535 | 0.346 | 1.50 | 2.00 |
|  | Hunan | 415.9 | 387.2 | 0.796 | 2.130 | 0.600 | 0.613 | 1.50 | 0.00 |
|  | Guangxi | 77.9 | 109.9 | 0.901 | 18.187 | 0.350 | 0.605 | 1.50 | 1.00 |
|  | Chongqing | 171.5 | 175.7 | 0.760 | 4.077 | 0.454 | 0.582 | 1.75 | 1.00 |
|  | Sichuan | 528.9 | 677.6 | 0.799 | 18.426 | 0.647 | 0.520 | 0.25 | 2.00 |
|  | Guizhou | 62.1 | 44.1 | 1.069 | 2.713 | -0.087 | 0.333 | 2.75 | 2.00 |
| Other | Hainan | 70.1 | 71.7 | 2.633 | 262.774 | -0.132 | -0.103 | 1.50 | NA^#^ |
|  | Yunnan | 288.6 | 500.9 | 0.680 | 13.637 | 0.450 | 0.322 | 1.75 | 1.00 |
|  | Tibet | 146.3 | 54.1 | 2.150 | 15.648 | 0.473 | 0.364 | 0.33 | 2.00 |
|  | Shaanxi | 82.7 | 128.8 | 0.665 | 3.024 | 0.873 | 0.366 | 0.50 | 1.50 |
|  | Gansu | 370.0 | 285.1 | 1.916 | 4.642 | 0.661 | 0.891 | 0.25 | 1.00 |
|  | Qinghai | 88.5 | 27.2 | 2.112 | 0.883 | 0.596 | 0.738 | 0.33 | 1.50 |
|  | Ningxia | 88.1 | 50.2 | 1.801 | 1.489 | 0.771 | 0.430 | 0.67 | 1.50 |
|  | Xinjiang | 1287.7 | 820.7 | 3.000 | 32.769 | 0.249 | 0.825 | 1.50 | 1.50 |

^#^Peak time difference was not calculated because the maximum monthly incidence for Hainan was too low (2 cases), and the observed peak timing could not be determined.
