# Supplementary material for: Rural-to-urban migrant worker mobility shaped measles epidemics in China
Source: PLoS Comput Biol. 2026 Apr 10;22(4):e1014182. doi: 10.1371/journal.pcbi.1014182 (PMC13170960; doi:10.1371/journal.pcbi.1014182)
Supplement: S2 Table — Under alternative scenario #1, daily mobility flow volumes for each corresponding day within each national holiday or inter-holiday period were set to the minimum observed in the original 2015–2019 mobility data; under alternative scenario #2, the flow volumes were set to the maximum observed. r: correlation coefficient between the simulated incidence time series under an alternative scenario and the base scenario; incidence difference: relative difference in cumulative incidence over 2005–2014 for an alternative scenario compared with the base scenario. (DOCX) [file pcbi.1014182.s015.docx]

**S2 Table.** Mobility sensitivity analysis comparing the two alternative mobility scenarios with the base scenario. Under alternative scenario #1, daily mobility flow volumes for each corresponding day within each national holiday or inter-holiday period were set to the minimum observed in the original 2015–2019 mobility data; under alternative scenario #2, the flow volumes were set to the maximum observed. *r*: correlation coefficient between the simulated incidence time series under an alternative scenario and the base scenario; incidence difference: relative difference in cumulative incidence over 2005–2014 for an alternative scenario compared with the base scenario.

| PLAD | Alternative scenario #1 | | Alternative scenario #2 | |
| --- | --- | --- | --- | --- |
|  | *r* | Incidence difference | *r* | Incidence difference |
| Beijing | 0.999 | 0.078 | 0.999 | 0.043 |
| Tianjin | 0.992 | 0.200 | 0.994 | 0.169 |
| Shanghai | 0.999 | 0.042 | 1.000 | 0.041 |
| Jiangsu | 0.998 | 0.116 | 0.997 | 0.118 |
| Zhejiang | 0.999 | 0.072 | 0.999 | 0.064 |
| Fujian | 0.997 | 0.111 | 0.997 | 0.115 |
| Guangdong | 0.999 | 0.074 | 0.998 | 0.060 |
| Hebei | 0.999 | 0.086 | 0.998 | 0.089 |
| Shanxi | 0.999 | 0.067 | 0.999 | 0.050 |
| Inner Mongolia | 0.999 | 0.059 | 0.999 | 0.039 |
| Liaoning | 0.999 | 0.050 | 1.000 | 0.029 |
| Jilin | 0.996 | 0.135 | 0.997 | 0.134 |
| Heilongjiang | 0.998 | 0.064 | 0.999 | 0.024 |
| Anhui | 0.998 | 0.053 | 0.999 | 0.050 |
| Jiangxi | 0.999 | 0.068 | 0.999 | 0.064 |
| Shandong | 0.999 | 0.066 | 0.999 | 0.052 |
| Henan | 0.998 | 0.112 | 0.998 | 0.101 |
| Hubei | 0.999 | 0.059 | 0.999 | 0.050 |
| Hunan | 0.999 | 0.047 | 0.998 | 0.033 |
| Guangxi | 0.996 | 0.122 | 0.998 | 0.085 |
| Chongqing | 0.998 | 0.075 | 0.999 | 0.057 |
| Sichuan | 0.997 | 0.106 | 0.997 | 0.100 |
| Guizhou | 0.999 | 0.052 | 0.999 | 0.060 |
| Hainan | 0.998 | 0.081 | 0.999 | 0.038 |
| Yunnan | 1.000 | 0.005 | 1.000 | 0.014 |
| Tibet | 1.000 | 0.019 | 1.000 | 0.020 |
| Shaanxi | 0.996 | 0.119 | 0.996 | 0.120 |
| Gansu | 0.999 | 0.018 | 0.999 | 0.041 |
| Qinghai | 0.996 | 0.074 | 0.996 | 0.094 |
| Ningxia | 0.998 | 0.107 | 0.999 | 0.084 |
| Xinjiang | 0.999 | 0.028 | 0.999 | 0.029 |
