# Supplementary material for: Rural-to-urban migrant worker mobility shaped measles epidemics in China
Source: PLoS Comput Biol. 2026 Apr 10;22(4):e1014182. doi: 10.1371/journal.pcbi.1014182 (PMC13170960; doi:10.1371/journal.pcbi.1014182)
Supplement: S3 Table — (DOCX) [file pcbi.1014182.s016.docx]

**S3 Table.** Relative differences in cumulative incidence between the four counterfactual scenarios and the baseline in origin PLADs and in PLADs that were neither host nor origin.

| Type | PLAD | Counterfactual scenario | | | |
| --- | --- | --- | --- | --- | --- |
|  |  | No travelers | No case importation | Matching population susceptibility | Pre-migration vaccination (100%) |
| Origin | Hebei | 2.0% (-21.5%, 28.8%) | -20.4% (-39.5%, 3.2%) | -15.0% (-36.2%, 9.3%) | -34.9% (-50.2%, -16.8%) |
|  | Shanxi | 4.1% (-27.5%, 46.8%) | -2.3% (-32.5%, 36.9%) | -1.6% (-31.7%, 34.0%) | -9.3% (-36.1%, 24.3%) |
|  | Inner Mongolia | 2.1% (-28.8%, 38.4%) | -2.1% (-29.6%, 35.5%) | 0.4% (-29.9%, 37.8%) | -8.1% (-34.2%, 24.5%) |
|  | Liaoning | -1.3% (-30.4%, 37.3%) | -1.4% (-32.7%, 36.7%) | -0.7% (-30.2%, 38.6%) | -6.7% (-35.8%, 28.5%) |
|  | Jilin | -4.4% (-31.3%, 31.8%) | -0.7% (-29.6%, 36.5%) | -0.3% (-28.4%, 36.5%) | -3.0% (-31.7%, 31.0%) |
|  | Heilongjiang | 1.2% (-28.4%, 39.8%) | -10.4% (-38.5%, 28.0%) | -7.9% (-36.1%, 29.0%) | -21.3% (-44.4%, 9.6%) |
|  | Anhui | 1.3% (-29.1%, 39.7%) | -34.5% (-65.1%, -0.7%) | -7.0% (-43.2%, 34.0%) | -69.9% (-79.6%, -57.7%) |
|  | Jiangxi | -1.5% (-26.2%, 28.7%) | -50.7% (-65.5%, -32.1%) | -21.1% (-43.3%, 5.8%) | -71.9% (-78.6%, -64.0%) |
|  | Shandong | 1.1% (-23.1%, 29.6%) | -19.0% (-40.6%, 7.2%) | -14.9% (-36.0%, 11.5%) | -29.0% (-47.8%, -6.7%) |
|  | Henan | -0.9% (-22.1%, 24.3%) | -36.3% (-53.1%, -16.9%) | -21.4% (-40.7%, 0.2%) | -59.1% (-69.0%, -48.1%) |
|  | Hubei | -2.7% (-27.0%, 26.5%) | -41.0% (-59.7%, -18.1%) | -21.8% (-44.4%, 5.8%) | -65.4% (-75.1%, -54.4%) |
|  | Hunan | -2.2% (-24.2%, 24.8%) | -21.7% (-42.6%, 6.4%) | -2.5% (-26.8%, 28.0%) | -61.8% (-71.2%, -49.8%) |
|  | Guangxi | -3.1% (-26.5%, 24.6%) | -47.1% (-62.6%, -28.0%) | -23.0% (-43.0%, 3.3%) | -68.7% (-76.7%, -58.7%) |
|  | Chongqing | 5.9% (-24.3%, 44.7%) | -2.3% (-30.0%, 34.0%) | 1.7% (-28.5%, 40.3%) | -13.2% (-39.0%, 20.1%) |
|  | Sichuan | 3.7% (-23.7%, 36.0%) | -35.4% (-54.6%, -13.8%) | -16.3% (-40.9%, 12.7%) | -62.6% (-73.2%, -50.5%) |
|  | Guizhou | 2.4% (-21.6%, 30.6%) | -57.2% (-68.4%, -45.4%) | -40.7% (-55.7%, -24.7%) | -65.6% (-73.5%, -56.3%) |
| Other | Hainan | 0.3% (-24.9%, 32.3%) | 1.5% (-24.1%, 31.2%) | -0.1% (-24.4%, 28.1%) | 2.3% (-22.6%, 31.9%) |
|  | Yunnan | 4.1% (-4.6%, 13.7%) | 0.2% (-8.5%, 9.3%) | 0.0% (-8.0%, 8.8%) | 0.3% (-8.2%, 9.0%) |
|  | Tibet | -3.9% (-22.9%, 18.2%) | 0.7% (-20.1%, 23.3%) | 0.5% (-18.4%, 23.4%) | 1.9% (-18.3%, 25.5%) |
|  | Shaanxi | -12.7% (-31.5%, 11.0%) | 0.4% (-23.2%, 27.4%) | 2.2% (-19.6%, 27.6%) | 3.5% (-18.6%, 31.4%) |
|  | Gansu | 4.1% (-14.2%, 26.0%) | -0.1% (-17.3%, 20.1%) | -0.1% (-17.8%, 19.3%) | -3.8% (-20.8%, 15.2%) |
|  | Qinghai | 2.0% (-28.0%, 39.1%) | 0.7% (-27.4%, 36.7%) | 1.5% (-26.4%, 37.1%) | -4.6% (-31.4%, 28.8%) |
|  | Ningxia | -4.3% (-30.8%, 28.2%) | -0.1% (-27.2%, 35.5%) | -0.1% (-27.1%, 32.7%) | -0.2% (-27.7%, 35.3%) |
|  | Xinjiang | 2.9% (-13.0%, 21.4%) | 0.4% (-15.3%, 17.3%) | 0.8% (-13.8%, 18.4%) | 1.1% (-13.8%, 19.0%) |
