# Supplementary material for: Rural-to-urban migrant worker mobility shaped measles epidemics in China
Source: PLoS Comput Biol. 2026 Apr 10;22(4):e1014182. doi: 10.1371/journal.pcbi.1014182 (PMC13170960; doi:10.1371/journal.pcbi.1014182)
Supplement: S4 Table — (DOCX) [file pcbi.1014182.s017.docx]

**S4 Table.** Relative differences in cumulative incidence between the “pre-migration vaccination” scenario at different coverage levels and the baseline in China and individual PLADs.

| Type | China or PLAD | “Pre-migration vaccination” scenario | | | |
| --- | --- | --- | --- | --- | --- |
|  |  | 100% coverage | 75% coverage | 50% coverage | 25% coverage |
| China | China | -50.9% (-54.2%, -47.2%) | -47.2% (-50.8%, -43.4%) | -43.0% (-46.9%, -39.1%) | -33.0% (-37.4%, -28.0%) |
| Host | Beijing | -79.1% (-82.2%, -76.0%) | -71.5% (-75.9%, -66.7%) | -65.6% (-70.7%, -60.5%) | -52.9% (-59.4%, -45.7%) |
|  | Tianjin | -83.9% (-86.8%, -80.8%) | -78.0% (-82.1%, -73.7%) | -74.0% (-79.0%, -68.5%) | -59.5% (-67.3%, -51.0%) |
|  | Shanghai | -73.7% (-78.1%, -69.0%) | -68.7% (-73.1%, -63.7%) | -62.3% (-68.0%, -56.3%) | -48.6% (-57.0%, -39.1%) |
|  | Jiangsu | -84.0% (-86.6%, -81.3%) | -80.3% (-85.9%, -75.9%) | -75.2% (-83.1%, -69.6%) | -58.9% (-71.0%, -48.6%) |
|  | Zhejiang | -82.3% (-84.9%, -79.4%) | -78.1% (-81.3%, -74.6%) | -72.9% (-77.1%, -68.5%) | -59.3% (-66.4%, -51.7%) |
|  | Fujian | -84.8% (-88.1%, -81.1%) | -77.6% (-83.6%, -71.4%) | -70.2% (-79.1%, -61.1%) | -56.5% (-68.6%, -43.5%) |
|  | Guangdong | -89.2% (-91.0%, -87.0%) | -85.9% (-88.3%, -83.5%) | -79.2% (-82.6%, -75.6%) | -60.6% (-67.9%, -51.5%) |
| Origin | Hebei | -34.9% (-50.2%, -16.8%) | -32.1% (-49.3%, -13.2%) | -28.3% (-45.4%, -8.7%) | -20.8% (-38.9%, -0.3%) |
|  | Shanxi | -9.3% (-36.1%, 24.3%) | -7.9% (-35.3%, 26.7%) | -5.6% (-32.6%, 29.9%) | -3.8% (-32.5%, 32.5%) |
|  | Inner Mongolia | -8.1% (-34.2%, 24.5%) | -6.9% (-33.4%, 24.0%) | -5.0% (-30.5%, 24.6%) | -3.2% (-28.3%, 24.7%) |
|  | Liaoning | -6.7% (-35.8%, 28.5%) | -6.9% (-33.8%, 30.5%) | -6.1% (-32.8%, 29.2%) | -4.2% (-31.7%, 31.9%) |
|  | Jilin | -3.0% (-31.7%, 31.0%) | -2.4% (-29.9%, 31.7%) | -2.2% (-28.0%, 29.3%) | -0.8% (-29.3%, 33.7%) |
|  | Heilongjiang | -21.3% (-44.4%, 9.6%) | -18.2% (-42.4%, 11.6%) | -15.7% (-39.1%, 14.0%) | -9.8% (-35.9%, 21.1%) |
|  | Anhui | -69.9% (-79.6%, -57.7%) | -65.8% (-78.3%, -50.9%) | -58.2% (-74.9%, -38.3%) | -41.6% (-62.0%, -15.8%) |
|  | Jiangxi | -71.9% (-78.6%, -64.0%) | -68.0% (-75.8%, -58.8%) | -61.1% (-70.6%, -50.3%) | -44.2% (-57.9%, -27.7%) |
|  | Shandong | -29.0% (-47.8%, -6.7%) | -26.2% (-45.3%, -2.2%) | -22.9% (-42.7%, 2.5%) | -16.5% (-37.5%, 10.0%) |
|  | Henan | -59.1% (-69.0%, -48.1%) | -55.2% (-65.2%, -43.8%) | -48.4% (-59.4%, -34.4%) | -35.1% (-49.3%, -18.2%) |
|  | Hubei | -65.4% (-75.1%, -54.4%) | -61.8% (-71.8%, -49.3%) | -54.1% (-66.7%, -38.6%) | -38.8% (-56.1%, -19.9%) |
|  | Hunan | -61.8% (-71.2%, -49.8%) | -56.3% (-67.4%, -43.4%) | -47.4% (-61.6%, -30.8%) | -31.8% (-48.9%, -9.2%) |
|  | Guangxi | -68.7% (-76.7%, -58.7%) | -64.8% (-73.7%, -52.5%) | -57.1% (-68.1%, -42.3%) | -40.8% (-55.6%, -20.8%) |
|  | Chongqing | -13.2% (-39.0%, 20.1%) | -11.1% (-35.8%, 18.7%) | -8.4% (-32.5%, 23.5%) | -4.9% (-31.3%, 29.4%) |
|  | Sichuan | -62.6% (-73.2%, -50.5%) | -57.3% (-68.8%, -42.5%) | -50.5% (-63.1%, -35.2%) | -35.5% (-52.3%, -14.3%) |
|  | Guizhou | -65.6% (-73.5%, -56.3%) | -61.5% (-70.0%, -52.7%) | -56.0% (-65.6%, -45.9%) | -39.7% (-53.2%, -24.5%) |
| Other | Hainan | 2.3% (-22.6%, 31.9%) | 2.2% (-22.9%, 32.0%) | -0.6% (-23.7%, 28.7%) | 2.2% (-22.9%, 32.0%) |
|  | Yunnan | 0.3% (-8.2%, 9.0%) | -0.2% (-8.0%, 8.1%) | 1.2% (-7.4%, 10.8%) | -0.2% (-8.0%, 8.1%) |
|  | Tibet | 1.9% (-18.3%, 25.5%) | 2.3% (-24.6%, 36.9%) | 1.5% (-26.2%, 37.9%) | 2.3% (-24.6%, 36.9%) |
|  | Shaanxi | 3.5% (-18.6%, 31.4%) | 4.2% (-14.0%, 25.3%) | 2.9% (-14.2%, 23.6%) | 4.2% (-14.0%, 25.3%) |
|  | Gansu | -3.8% (-20.8%, 15.2%) | -2.2% (-15.8%, 12.6%) | -2.0% (-15.2%, 12.0%) | -2.2% (-15.8%, 12.6%) |
|  | Qinghai | -4.6% (-31.4%, 28.8%) | -2.7% (-27.8%, 28.5%) | -2.5% (-26.5%, 27.5%) | -2.7% (-27.8%, 28.5%) |
|  | Ningxia | -0.2% (-27.7%, 35.3%) | -2.3% (-24.0%, 24.5%) | -0.3% (-23.7%, 29.6%) | -2.3% (-24.0%, 24.5%) |
|  | Xinjiang | 1.1% (-13.8%, 19.0%) | 1.4% (-11.5%, 16.0%) | 1.5% (-11.5%, 16.9%) | 1.4% (-11.5%, 16.0%) |
