# Supplementary material for: Rural-to-urban migrant worker mobility shaped measles epidemics in China
Source: PLoS Comput Biol. 2026 Apr 10;22(4):e1014182. doi: 10.1371/journal.pcbi.1014182 (PMC13170960; doi:10.1371/journal.pcbi.1014182)
Supplement: S5 Table — (DOCX) [file pcbi.1014182.s018.docx]

**S5 Table.** Values of constant model parameters.

| Parameter | Value |
| --- | --- |
| Proportion of migrant workers who remain in host PLADs during CNY, $\theta$ | 0.5 (1) |
| Employment rate of migrant workers, $\eta$ | 0.7 (2) |
| Average duration of stay as migrant workers, $L$ (year) | 3 (3) |
| Duration of return migration to origin PLADs before CNY, $T_{preCNY}$ (week) | 6 |
| Duration of CNY holiday, $T_{CNY}$ (week) | 1 |
| Duration of migration to host PLADs after CNY, $T_{postCNY}$ (week) | 6 |
| Duration of job-seeking period of migrant workers in host PLADs, $T_{job seek}$ (week) | 8 |

**References**

1. Qiu G. National Bureau of Statistics: Total number of rural-to-urban migrant workers in China reached 225.42 million by the end of 2008 (in Chinese): China News Service; 2009. Available from: <https://www.chinanews.com/cj/kong/news/2009/03-25/1616960.shtml>.

2. International Labour Office for China and Mongolia. Employment Policies Report—China: Promoting Decent Employment for Rural Migrant Workers. Geneva, Switzerland: International Labour Office; 2011. Available from: <https://www.ilo.org/sites/default/files/wcmsp5/groups/public/@asia/@ro-bangkok/@ilo-beijing/documents/publication/wcms_184814.pdf>.

3. Liu B, Feng C, Shen B. Characteristics of intra-urban migration of rural migrant workers in Beijing (in Chinese). Urban Studies. 2012;19(5):72–6.
