# Supplementary material for: Rural-to-urban migrant worker mobility shaped measles epidemics in China
Source: PLoS Comput Biol. 2026 Apr 10;22(4):e1014182. doi: 10.1371/journal.pcbi.1014182 (PMC13170960; doi:10.1371/journal.pcbi.1014182)
Supplement: S6 Table — (DOCX) [file pcbi.1014182.s019.docx]

**S6 Table.** Initial ranges of model state variables and parameters.

| State variable and parameter | | Range |
| --- | --- | --- |
| State variable | Susceptible population ($S$) | Sampled from a uniform distribution with the minimum set to the population model estimate -0.015 (i.e., 1.5% of the population size) and the maximum set to the population model estimate +0.015 (S10 Fig; see details in S1 Text “Model initialization”) |
|  | Exposed population ($E$) | Initial observed incidence adjusted by reporting rate, distributed among subpopulations assuming equal prevalence, and sampled from a negative binomial distribution |
|  | Infectious population ($I$) | Initial observed incidence adjusted by reporting rate, distributed among subpopulations assuming equal prevalence, and sampled from a negative binomial distribution |
| Parameter | Basic reproductive number based on contact ($R_{0,cont}$) | [8, 20] (1) |
|  | Minimum basic reproductive number in an absolute humidity and temperature-forced model ($R_{0,min,clim}$) | [6, 15] (1) |
|  | Difference between maximum and minimum basic reproductive numbers in an absolute humidity and temperature-forced model ($R_{0,diff,clim}$) | [4, 18] (1) |
|  | Transmission rate within subpopulation, relative to $\beta_{1}$ $(\beta_{1}^{'}$) | 1 |
|  | Transmission rate between subpopulations originally from different PLADs, relative to $\beta_{1}$ $(\beta_{2}^{'}$) | $[0,\frac{0.4}{a}]$ (the upper bound (sum of the element (1,2) in S18 Eq) was set as 0.4, estimated by the largest proportion of migrant workers among PLADs, and by assuming homogeneous mixing among local and migrant worker subpopulations) |
|  | Transmission rate between subpopulations originally from a same PLAD, relative to $\beta_{1}$ $(\beta_{3}^{'}$) | $[0,\frac{0.2}{b}]$ (the upper bound (sum of the element (1,3) in S18 Eq) was set as 0.2, estimated by the largest proportion of returning migrant workers among PLADs, and by assuming the local subpopulation had the same contact rates within itself and with the returning migrant worker subpopulations) |
|  | Mixing exponent within subpopulation ($m_{1}$) | [0.85, 1] |
|  | Mixing exponent between subpopulations originally from different PLADs ($m_{2}$) | [0.4, 1] |
|  | Mixing exponent between subpopulations originally from a same PLAD ($m_{3}$) | [0.4, 1] |
|  | Latent period ($Z$) | [7, 9] (1) |
|  | Infectious period ($D$) | [4, 6] (1) |
|  | Reporting rate ($\rho$) | Time series susceptible–infected–recovered model with inputs of incidence, birth, and total population data during 2005–2008 (1-3) |

**References**

1. Wang P, Chen J, Zhang W, Wang Y, Yang W. Modeling the influences of climate conditions on measles transmission in China. Epidemiology and Infection. 2025;153:e110.

2. Finkenstädt BF, Grenfell BT. Time series modelling of childhood diseases: A dynamical systems approach. Journal of the Royal Statistical Society: Series C (Applied Statistics). 2000;49(2):187–205.

3. Becker AD, Grenfell BT. tsiR: An R package for time-series Susceptible-Infected-Recovered models of epidemics. PLOS One. 2017;12(9):e0185528.
